# Rhizosphere Fungal Community Responses to Nitrogen Addition Reflect Genotypic Versus Trait Variation in the Marsh Sedge *Schoenoplectus americanus* Over Time

**DOI:** 10.1111/mec.70588

**Published:** 2026-10-14

**Authors:** Yue Liu, Michael J. Blum, Candice Y. Lumibao

**Affiliations:** ^1^ Department of Life Sciences Texas A&M University – Corpus Christi Corpus Christi Texas USA; ^2^ Department of Ecology & Evolution University of Tennessee – Knoxville Knoxville Tennessee USA

**Keywords:** eco‐evolutionary dynamics, fungal guild diversity, G × E interactions, global change, plant‐microbe interactions, resurrection ecology

## Abstract

There is growing recognition that shifting environmental conditions can alter the nature and potential benefits of plant‐microbe interactions. While it is well established that interactions with fungi can benefit plants under environmental stress, little is known about how plant exposure responses correspond to different factors that might shape fungal communities, particularly over time. Using a resurrection ecology approach, we examined the relative influence of contrasting environmental conditions and intraspecific variation in the foundational marsh sedge *Schoenoplectus americanus* on rhizosphere fungal communities. Cohorts of ancestral plants germinated from 100+ year old seeds and modern plants were grown under low and high nitrogen (N) concentration in a common‐garden experiment. Fungal communities were characterized according to plant presence as well as genotypic and associated trait variation. The study revealed that the influence of *S. americanus* on fungal communities differed between the nitrogen regimes. Under low N, fungal taxonomic, but not guild, diversity was higher in rhizosphere soils than unplanted controls. Plant effects were, however, diminished under high N conditions. We also found that heritable traits had limited influence on fungal communities, whereas both taxonomic and guild diversity and composition reflected genotypic variation and G × E interactions. Cohorts of ancestral and modern plants also recruited distinct fungal communities depending on N conditions. These results indicate that shifts in plant‐fungal associations over time are contingent on prevailing environmental conditions. Our findings also indicate that N‐driven changes in plant–fungal interactions likely alter ecosystem processes that are central to saltmarsh resilience to shifting environmental conditions.

## 1 Introduction

Plants interact with a multitude of fungi, including those that inhabit the rhizosphere (i.e., the zone within ~2 mm surrounding roots). Shifting environmental conditions can alter the nature of and potential advantages arising from plant‐microbe interactions, including organismal and community persistence (Trivedi et al. 2022). Recent studies have illustrated, for example, that soil microbes can mediate plant responses to environmental stressors such as salinity and drought by increasing plant fitness (e.g., Trivedi et al. 2022; Lumibao et al. 2022). Likewise, plants can influence the responses of associated fungi to environmental pressures by modifying community composition and functions (e.g., Fitzpatrick et al. 2018; Lumibao et al. 2025). However, the nature and strength of plant‐fungal interactions under prevailing environmental conditions can be subject to a range of different factors including heritable traits and genotypic variations in plants. Associations are also likely to shift over time (i.e., within and across generations of plants) as the environment changes (Lumibao et al. 2022). Thus, gaining further insights into how different factors influence plant‐fungal associations over time could offer a strong basis for predicting outcomes of ongoing environmental change.

Fungal communities are subject to the influence of plants with which they associate. It is well established that microbial associations differ among plant species (Fitzpatrick et al. 2018). It also appears that intraspecific plant variation can exert as much influence as interspecific differences on microbes, including those in the rhizosphere (e.g., Lumibao et al. 2020; Negre Rodríguez et al. 2025) and endosphere (i.e., inside plant tissues) (Lumibao et al. 2025). For example, different genotypes of the marsh plant, *Spartina alterniflora* , associate with distinct rhizosphere (Lumibao et al. 2020) and endophytic communities (Hanley et al. 2025). Trait differences among genotypes might alter root‐associated resources and soil conditions (e.g., carbon availability) that influence fungal assemblages (Zhalnina et al. 2018; Broeckling et al. 2008). Large root biomass, for example, tends to release high quantities of root exudates (e.g., sugars and amino acids) that favour growth of specific taxa (Broeckling et al. 2008). Genotypic variation in tissue chemistry (e.g., lignin and nitrogen content) can also lead to differences in substrate composition, promoting different functional fungal guilds (e.g., decomposers) without changing taxonomic diversity (Bhatnagar et al. 2018). Accordingly, rhizosphere microbes are sometimes considered to be an ‘extended root phenotype’ of associated plants (i.e., a manifestation of plant gene effects (Dawkins 1982; de la Fuente Cantó et al. 2020)) that can be subject to selective pressures.

Shifts in heritable variation in phenotypic traits over time can alter how plants interact with microbial communities (Lumibao 2026). With exposure to environmental pressures, plant traits like root architecture and resource allocation can evolve such that they differ between parent and descendant populations (Vahsen, Blum, et al. 2023; Christie et al. 2023). For example, ancestral plants (grown from century‐old seeds) and modern descendant genotypes of *Schoenoplectus americanus* exhibit differences in heritable traits, with modern genotypes exhibiting reduced belowground biomass allocation, potentially reflecting selection associated with increased anthropogenic nitrogen inputs (Vahsen, Blum, et al. 2023). Microbial mediation of plant responses to abiotic stress also differed between ancestral and descendant cohorts of *S. americanus* genotypes (Lumibao et al. 2022). To date, most other studies have relied on space‐for‐time substitutions (e.g., Evans et al. 2025) to infer how associations shift over time or across plant generations, but this approach might not necessarily capture representative characterizations of dynamic plant‐microbe associations. Further understanding might thus be gained through additional study involving approaches like resurrection ecology that can offer insight on the extent to which plant genotypic and heritable trait variations shape fungal communities over time.

Prevailing environmental conditions can also determine the strength and direction of plant‐fungal associations. Under a relatively benign environment, both plant and fungal communities may establish stable or positive associations. However, the nature of associations may shift when environmental conditions change, such as when nitrogen (N) levels rise. It has been shown, for example, that the relative influence of plants on rhizosphere fungal communities can weaken because abiotic stress can act as a strong selective pressure (Kivlin et al. 2013). N enrichment can also influence rhizosphere fungi directly through abiotic filtering and indirectly via plant‐mediated responses to greater nutrient availability. For instance, elevated N may favour nutrient‐responsive fungal taxa while also altering root‐derived resources through shifts in biomass allocation (Kearns et al. 2019; Zhalnina et al. 2018). Notably, the influence of plants on fungal communities may fluctuate over time under a variable environmental regime.

Coastal marshes provide opportunities for evaluating the nature of plant‐fungal associations under conditions of environmental stress including nutrient loading, which can elicit biotic responses that weaken ecosystem integrity (Bardgett and Van Der Putten 2014; Deegan et al. 2012). Coastal marshes are often dominated by foundational plants that are sensitive to N enrichment, including the C_3_ perennial sedge *Schoenoplectus americanus* (Vahsen, Blum, et al. 2023) which is widespread across the North Atlantic Gulf and Atlantic coast marshes. There is good reason to think that responses of *S. americanus* to nutrient loading affect associated fungal communities. Previous work has shown that *S. americanus* exhibits considerable heritable variation in belowground traits such as root biomass and root‐to‐shoot ratio (Vahsen, Blum, et al. 2023) in response to abiotic stress and soil microbial inoculation (Lumibao et al. 2022). Further study might thus offer greater understanding of how genotypic and trait variations in *S. americanus* influence fungal communities under different N conditions, and in turn, coastal marsh ecosystem resilience to ongoing anthropogenic pressures.

Here, we examine how *S. americanus* can shape rhizosphere soil fungal communities under contrasting nutrient conditions. To do so, we examined how intraspecific genotypic and heritable trait variation in *S. americanus* affects the function and structure of rhizosphere fungal communities under distinct N treatments. We tested three hypotheses. First, we examined whether (**H1**) plant presence, regardless of plant genotype, alters fungal taxonomic and functional/guild diversity in response to N addition. Alternatively, plant presence might change fungal community structure independently of N addition. Next, we hypothesized (**H2a**) that taxonomic and functional variation in fungal communities is driven by plant trait variation among *S. americanus* genotypes, including between ancestral and modern descendant genotypes (**H2b**). Finally, we hypothesized that (**H3**) plant‐fungi associations are subject to genotype‐by‐environment (G × E) interactions (*sensu* the ‘extended root phenotype hypothesis,’ Dawkins 1982) (Figure 1).

**FIGURE 1:**
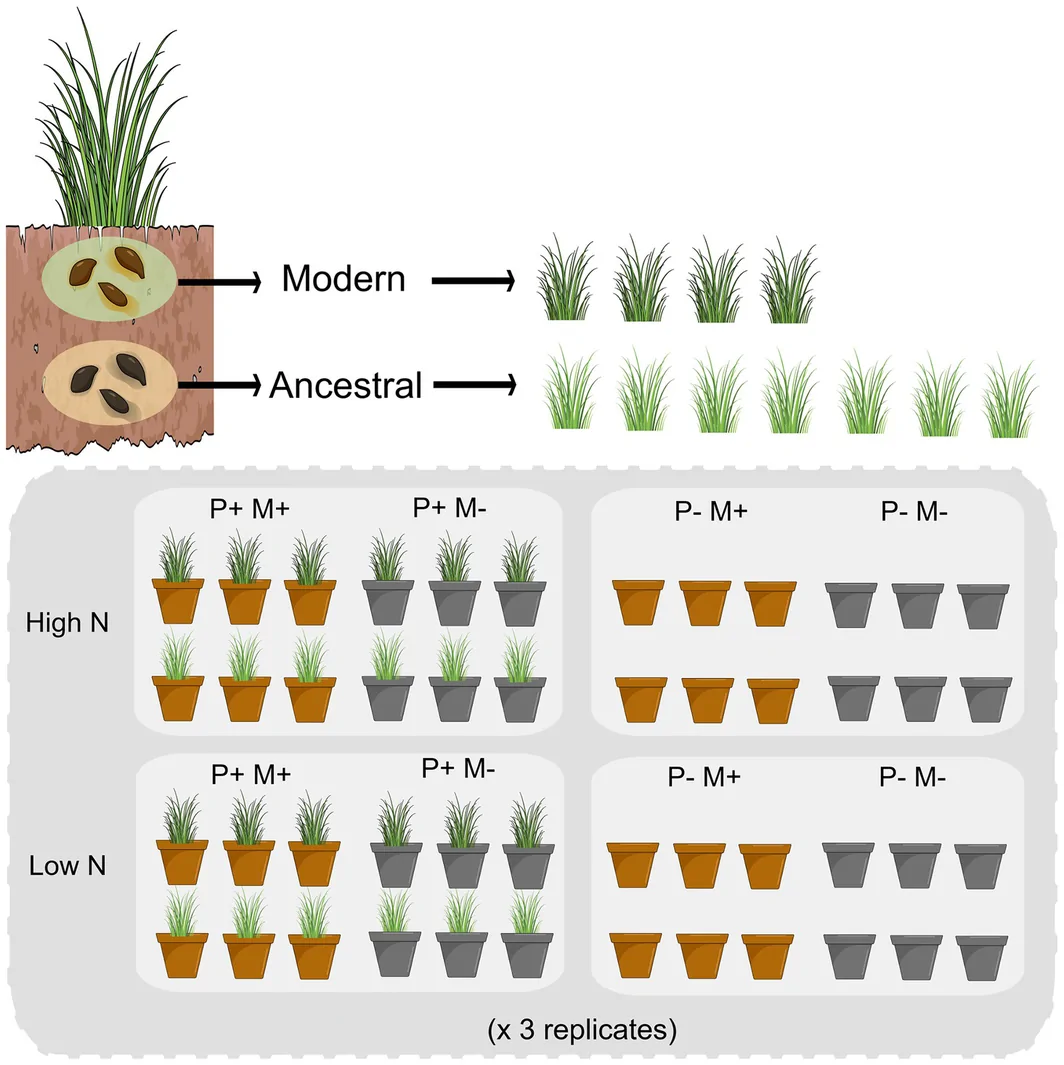
Experimental design. The greenhouse exposure experiment consisted of 11 ancestral and modern genotypes, under two plant treatments (with plant: P+M+ and no plant: P−M+) and two corresponding controls (plant control (P+M−) and microbial control (P−M−)) subjected to two N levels (High N: 25 g m^−2^ year^−1^ NH_4_Cl; Low N: 5 g m^−2^ year^−1^ NH_4_Cl). Each treatment was replicated three times using clonal propagules (total *n* = 228). Figure created with Mind the Graph.

## 2 Methods

### 2.1 Study System and Seed Retrieval

Seeds of *S. americanus* were recovered from soil cores of time‐stratified seed banks from the Corn Island marsh in the Rhode River estuary of Chesapeake Bay (Lumibao et al. 2022; Vahsen, Blum, et al. 2023; Vahsen, Kleiner, et al. 2023). Soil cores were previously dated using ^210^Pb and ^137^Cs radioisotope methods (Ritchie 2001), with seed profiles and ages inferred according to the core stratigraphy (Summers et al. 2018; Vahsen, Blum, et al. 2023; Vahsen, Kleiner, et al. 2023). Recovered seeds were then germinated and grown in controlled environment chambers at the University of Tennessee, Knoxville (UTK). Germinated *S. americanus* were clonally propagated and genotyped for prior studies (Vahsen, Blum, et al. 2023) (see Supporting Information S1).

### 2.2 Experimental Design

We conducted a greenhouse experiment using clones of *Schoenoplectus americanus* derived from ancestral (*ca*. 100‐year‐old seeds, collected from > 16 cm depth) and modern descendant (upper < 4 cm) age cohorts (Figure 2) (Summers et al. 2018). We used a mix of background soil and field marsh soil that served as a source for marsh microbes (see Supporting Information S1). The background soil consisted of a 1:1 mixture of topsoil and sand, providing a consistent growth medium across treatments. All background soil was sterilized prior to the experiment by autoclaving at 121°C for 2 h, repeating the process after 24 h (Lumibao et al. 2022). Soil inoculum was taken from a marsh in Oso Bay near Corpus Christi (Texas, USA) due to logistical constraints including long‐distance transport of ‘live’ soil (more details in Supporting Information S1) from Chesapeake Bay. Although microbial communities might vary among marshes, this approach still enabled us to test the stated hypotheses. Marsh‐derived soil was immediately sieved following collection to manually remove plant debris and rhizomes. Half of the marsh soil was sterilized as described above, and the other half was kept intact with soil microbes.

**FIGURE 2:**
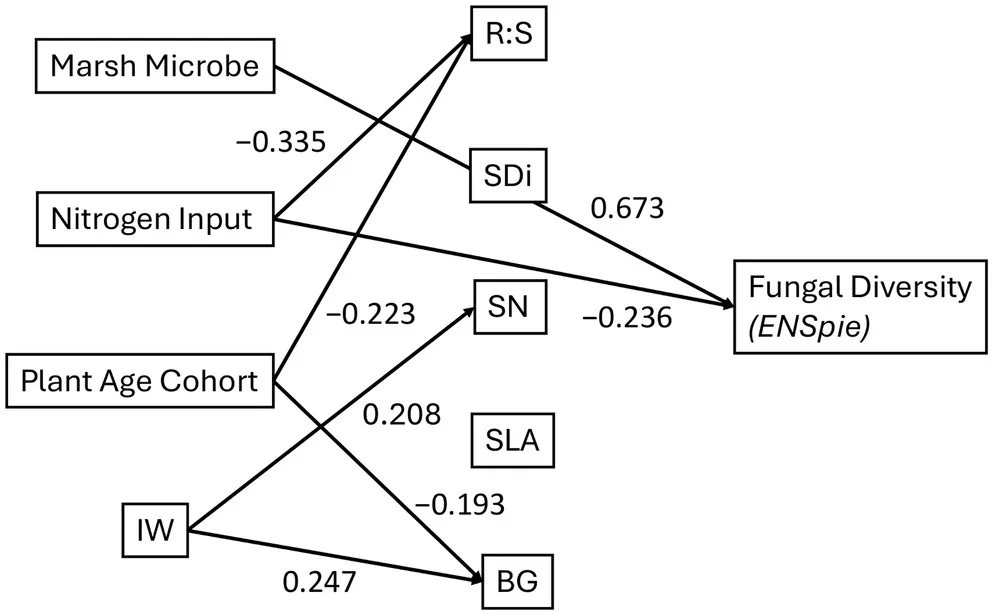
Path analyses. Structural equation model (SEM) describing factors influencing ASV‐level rhizosphere *ENSpie* diversity. The model quantifies direct and indirect effects of marsh microbe inoculation, N input, plant age cohort, and plant heritable traits on fungal diversity. Arrows represent significant causal paths (*p* < 0.05), and numbers indicate standardized path coefficients. Only significant relationships are depicted.

The factorial exposure experiment consisted of the following treatments: two plant treatments containing marsh microbes (with plants (P+) and no plant (P−)) at two N levels (low N: 5 g m^−2^ year^−1^ NH_4_Cl; high N: 25 g m^−2^ year^−1^ NH_4_Cl) (Langley et al. 2013). Two types of controls were included: (1) a plant control (P+M−) consisting of *S. americanus* grown in sterile background and marsh soil, and (2) a microbe control (P−M−) with no plants and sterile soil. Thus, the full plant–soil microbe (or PSM) treatments included four categories: P+M+ (plant with marsh microbes), P−M+ (no plant with marsh microbes), P+M− (plant with no microbes), and P−M− (no plant and no microbes). For P+M+ and P+M− treatments, 11 genotypes of *S. americanus* were used, including seven ancestral and four modern genotypes (three modern genotypes perished prior to transplanting). For each genotype, three sets of four replicate clones were established. Each pot (15 cm diameter, 60 cm height), hereafter ‘mesocosm’, received one clone or corresponding soil. Thus, there was a total of 228 mesocosms included in the experiment. Plants were allowed to establish for 2 weeks before the introduction of N. Plants were grown for 4 months, with water levels kept at 1 cm below the soil surface. Mesocosms were maintained under ambient natural light conditions, rotated biweekly to remove possible greenhouse effects, in a grow‐out facility at Texas A&M University AgriLife in Flour Bluff, Corpus Christi. All materials, including pots, watering sleeves, gloves, and tools, were sterilized with 20% bleach and 70% ethanol or UV light prior to use.

### 2.3 Plant Trait Measurement

Prior to transplanting clones, we recorded initial height (IH) and weight (IW) of all propagules. Plants were harvested at the end of the experiment, and a suite of traits was measured for each mesocosm: averaged stem height (SH), total number of living and dead stems (SN) at harvest, stem density (SD, stem number cm^−2^) calculated as the number of stems divided by the pot surface area (*πr* ^2^), and average stem diameter (SDi). We also measured specific leaf area (SLA) using a Li‐3100C area meter (LI‐COR Biosciences, Lincoln, NE, USA), calculated as total leaf area divided by total leaf dry mass. Aboveground (AG) and belowground (BG) biomass were dried at 65°C for three days, and the root‐to‐shoot ratio (R:S) was calculated as the ratio of AG and BG dry biomass.

### 2.4 Molecular Approaches

Soil samples were collected at the end of the experiment either directly from each pot (i.e., in mesocosms without plants) or from the soil attached to roots (i.e., rhizosphere soil, within a ~3 mm zone). Samples were immediately transferred to −80°C storage until further processing. Total genomic DNA was extracted from the soil using the DNeasy PowerSoil Kit (Qiagen, Germantown, MD, USA) following the manufacturer's guidelines. The extracted DNA was quantified using a Qubit 2.0 Fluorometer (Invitrogen, Carlsbad, CA, USA) and standardized to 10 ng/μL prior to PCR applications.

We amplified the Internal Transcribed Spacer (ITS) region using a two‐step process as described elsewhere (Lumibao et al. 2018, 2022). Briefly, the ITS2 region was amplified with the ITS1F and ITS2 primers modified with the TruSeq Adapter in triplicate reactions per sample (Gardes and Bruns 1993; White et al. 1990). The resulting triplicate reactions were then pooled for each sample and 1 μL of the PCR reactions was used for an indexing PCR. Indexing PCRs included seven unique dual barcodes for each sample to create DNA libraries. Further details can be found in Supporting Information S1. DNA libraries were purified using the Qiaquick Purification Kit (Qiagen, Germantown, MD, USA) and pooled at 25 ng per sample. Sequencing was carried out on the Illumina MiSeq 2 × 250 platform by the Texas A&M AgriLife Genomics and Bioinformatics Service.

### 2.5 Bioinformatics Analysis

Sequence data were processed using the QIIME 2 v. 2024.2 (Bolyen et al. 2019). Only forward reads (R1) were used for downstream analyses. Primers were trimmed from raw sequences using the qiime cutadapt trim‐single command with default parameters (maximum error rate = 0.1) (Martin 2011). Quality assessment of the sequences was performed using qiime demux summarize, and denoising was conducted with DADA2 (Callahan et al. 2016) through the qiime dada2 denoise‐single command, generating amplicon sequence variants (ASVs). The denoising process included trimming primers, chimera detection and removal, trimming the first 10 bases, and truncating reads to 220 bases. Taxonomic classification of the ASVs was conducted using a pre‐trained Naïve Bayes classifier against the UNITE v.9 database (Abarenkov et al. 2023) implemented in QIIME 2. Our full bioinformatics workflow is described in Supporting Information S1. Fungal guild was assigned to the taxa using FUNGuild (Nguyen et al. 2016). Only guilds with “highly probable” and “probable” confidence levels were retained as recommended by Nguyen et al. (2016), with the rest of the taxa binned into unassigned guilds.

### 2.6 Statistical Analysis

To test H1, we first examined the overall influence of *S. americanus* presence on fungal communities regardless of genotype under different N conditions across all samples. Two *α*‐diversity metrics were calculated: the *ENSpie* (Effective Number of Species based on the Probability of Interspecific Encounter (Chase and Knight 2013)) and the Shannon diversity index. *ENSpie* provides a scale‐independent measure that is less sensitive to sampling effort and rare species (Chase and Knight 2013; Seabloom et al. 2019; Henning et al. 2021). In our data, *ENSpie* is positively correlated with Shannon's diversity (Pearson's *r* = 0.876, *p* = 0.001) across all samples, and accordingly, results based on each index were qualitatively similar. Thus, hereafter we focus on *ENSpie* results, though we report the Shannon diversity results in Supporting Information S2. Sequences were rarefied to *n* = 3576 prior to analyses.

Generalized linear model analyses (GLMs) were conducted to determine the influence of plant presence on fungal diversity (*α*‐diversity), with *ENSpie* diversity as the response variable. For the model, we separated the four PSM treatments into two treatment types: plant presence (P+/P−) and marsh microbe (M+/M−) treatments. Doing so allowed us to explicitly examine the magnitude of the effect of plant presence under different N regimes on marsh microbes, accounting for background microbes that might have accumulated over the course of the experiment. Thus, the model includes N input (high or low), plant presence (P+ or P−), marsh microbe (M+ or M−), and all interactions as fixed factors, with *ENSpie* as the response variable. Models were run in R with the glm function in the stats package (R Core Team 2025) using a Gamma distribution with log link and model significance assessed using Type II *F*‐tests based on Wald statistics. If factors were found significant, we explored treatment differences using estimated marginal means (EMMs) (emmeans; Lenth and Piaskowski 2026) with Tukey's adjustment.

To further quantify the magnitude of plant presence on fungal diversity (hereafter, “plant effect”), we calculated the plant effect response variable similar to Lumibao et al. (2022) and Petipas et al. (2021). To do so, we examined data from P+M+ and P−M+ treated pairs of soils containing marsh microbes, within specific N and plant treatments and clonal replicates of *S. americanus* genotypes. *ENSpie* diversity values of P−M+ (plant‐absent) mesocosms were subtracted from the values of corresponding P+M+ (plant‐present) mesocosms within specific treatments and blocks. Resulting values > 0 indicated the presence of plants increased fungal diversity (positive effect), around zero indicated no effect, and values < 0 indicated a negative effect where fungal diversity decreased with plant presence. To further account for background microbial communities, we conducted a separate stepwise analysis of P+ vs. P− pairs across all samples. Briefly, we first subtracted fungal diversity values of plant controls (i.e., plants with no marsh microbes; P+M−) from plants with marsh microbes (P+M+) (hereon, *Diff 1*) as well as the fungal diversity values of microbe controls (P−M−) from soil with microbes (P−M+) independent of plants (hereafter, “*Diff 2*”) within specific N treatment and replicates. We then subtracted *Diff 2* from *Diff 1*.

To examine compositional changes in fungal communities (*β*‐diversity) due to plant presence and N treatment, we conducted Permutational Multivariate Analysis of Variance (PERMANOVA) across all samples. Abundance‐weighted community dissimilarities were calculated using the Bray–Curtis index. The model included the Bray–Curtis dissimilarity matrix as the response variable, and plant treatment × marsh microbe treatment × N level as predictors. Analyses were performed using the adonis2 function in vegan (Oksanen et al. 2026) with 9999 permutations. To further gain perspectives on fungal community variation, we conducted distance‐based redundancy analysis (db‐RDA) on Bray–Curtis index using the capscale function in vegan, with model significance tested with permutation ANOVA and forward model selection conducted using adjusted *R* ^2^ criteria.

We evaluated the diversity and compositional differences in the abundances of different fungal guilds (i.e., lifestyle modes) to determine whether patterns observed at ASV level are mirrored by guild. ASVs were assigned to specific guilds (e.g., saprotroph and symbiotroph) based on their taxonomic classification in FUNGuild. Sequence counts were then summed according to guild. All unassigned ASVs were included in the analyses and grouped into a single ‘Unassigned’ category. GLM analyses, pairwise ‘plant effect’ and PERMANOVA analyses described above were conducted for guild data.

We used a subset of the data that included only the planted mesocosms (i.e., P+M+ and P+M−) to test the influence of plant genotypic and trait variation (H2a) across age cohorts on fungal communities (H2b). To test H2a, we used a multi‐model inference and model averaging approach (Grueber et al. 2011) given that there could be several similarly informative models due to correlations among plant traits. We first fit a global mixed‐effects model as the base model, using a Gamma (log link) distribution for *ENSpie*. Marsh microbe (P+M+ and P+M−), N input and their interaction, age cohort (ancestral vs. modern), and plant traits (SN, SDi, R:S, SLA, and BG) were considered as fixed effects and individual genotype as a random effect. Initial propagule weight was also included as a covariate. Plant traits were standardized (*z*‐scaled) with a mean of 0 and standard deviation of 1 to compare estimates among variables. We used the MuMIn::dredge function (Bartoń 2026) to fit all possible models, with the experimental treatments included in all models. We then used the model.avg function to estimate relative weighted importance of each predictor variable across the top model, set at ΔAICc ≤ 4 (Grueber et al. 2011). Model fit and assumptions were evaluated using residual diagnostics and simulation‐based tests implemented in DHARMa package (Hartig 2026).

We determined the effects of the PSM and nitrogen treatments, plant traits, and age cohort on fungal community compositions by performing PERMANOVA using the model *Y* = N * marsh microbe (P+M+ and P+M−) + plant traits (SDi, SN, R:S, SLA, BG) + IW + Age Cohort (where *Y* is the Bray–Curtis dissimilarity matrix). Adonis2 in the vegan package was conducted with 9999 permutations and marginal (Type II‐like) sums of squares (by = “margin”). To further quantify constrained community variation, we performed distance‐based redundancy analysis (dbRDA) with IW, plant traits, age cohort, and nitrogen input as predictors. Given that P+M− is the control with no initial marsh microbes (and therefore would naturally contain different microbial communities), we partitioned out the effect of marsh microbes to control for their effects. We fit a dbRDA with marsh microbe as a covariate, which removes associated variations, and thus only evaluates the unique contribution of traits, cohort, and nitrogen to constrained variation. db‐RDA was conducted using capscale() with Bray‐Curtis distances as the response matrix, and used forward model selection with 9999 permutations to identify significant predictors.

As fungal communities might be directly responding to nitrogen enrichment, we tested for direct and indirect effects (via plant traits) of nitrogen on fungal *α*‐diversity to determine causal pathways upon which N influences fungi. A multilevel path analysis (i.e., structural equation modelling, SEM) (Shipley 2009) was conducted using the package piecewiseSEM (Lefcheck 2016). The meta‐model consisted of combined direct and indirect (via plant traits) effects of N, marsh microbe, and age cohort (Figure 1). We first constructed separate linear models for each plant trait, with IW, age cohort, N input, and marsh microbe as predictors. Including genotype as a factor resulted in unstable variance estimates, therefore genotype was not retained for SEM. We then constructed a model for *ENSpie* with plant traits (indirect), age cohort (indirect), and N (direct) as predictor variables. Correlated residual error terms among plant traits were also specified to improve overall model fit, which was evaluated using Fisher's C statistic and associated chi‐square tests. For each pathway, standardized coefficients were extracted, along with model‐specific *R* ^2^ values, to assess the relative strength of predictors. Indirect effects were evaluated by multiplying relevant path coefficients. All analyses were also conducted at the guild level.

To identify fungal taxa associated with specific environmental and plant factors (e.g., age cohort), we conducted indicator species analyses on both ASV and guild data. Analyses were performed separately to test associations with N input (low vs. high), plant age cohort (ancestral vs. modern), plant genotype, and the interaction between N input and plant age cohort. Indicator species analyses were conducted with 9999 permutations using the multipatt function in the indicspecies package (De Cáceres et al. 2010; De Cáceres and Legendre 2009). Analyses were restricted to single‐group indicators (maximum order = 1).

Lastly, we conducted generalized linear mixed modelling (GLMMs) on all P+ samples to test H3 (i.e., G × E interactions), following the hypothesis that microbial associates are an extended phenotype of plants. We fitted GLMMs with N × marsh microbe treatments (P+M+ vs. P+M−) as predictor variables and fungal *ENSpie* diversity as the response variable (i.e., “trait”). Plant genotype nested within N treatment was included as a random effect. Similar analyses were conducted for ASV and guild diversity. We visualized reaction norms and calculated the best linear unbiased predictors (BLUPs) from the fitted linear mixed model to estimate the mean trait value of each genotype. Results were plotted in ggplot2 (Wickham 2016). As in H2, only the P+ mesocosms were included in the analyses (i.e., both P+M+ and the plant control P+M−).

All statistical analyses were performed in R version 4.5.2 (R Core Team 2025). Visualizations were generated using the ggplot2 package (Wickham 2016).

### 2.7 Testing for Potential Bias in Uneven Sampling

We conducted two different tests to determine the robustness of our results and to address potential bias of uneven number of ancestral versus modern genotypes. First, homogeneity of variances between ancestral and modern cohorts (seven ancestral vs. four modern genotypes) was assessed for each plant trait and fungal diversity using the leveneTest function from the car package (Fox et al. 2026). Second, we evaluated all possible subsamples of the larger cohort (Ancestral; *n* = 7 genotypes) to match the number of modern genotypes (*n* = 4), resulting in 35 unique genotype combinations. For each combination, we calculated genotype‐level means and then averaged these values within cohorts to ensure equal weighting of genotypes. A two‐sided binomial test was used to evaluate whether the observed frequency differed from the expectation under random chance (*p* = 0.5) (Christie et al. 2023). Results showed that differences between cohorts were not driven by unequal genotype representation. Levene's tests showed no evidence that variance differed between cohorts (all FDR‐adjusted *p* > 0.05). Similarly, all possible genotype combinations of ancestral genotypes to match the number of modern genotypes produced consistent results across comparisons.

## 3 Results

### 3.1 Summary Statistics

We obtained a total of 5,112,386 fungal sequences across 228 soil samples, binned into 4149 unique ASVs. At the phylum level, Ascomycota accounted for 78.5% of the sequences, followed by Basidiomycota (10.5%), Rozellomycota (2.2%), Aphelidiomycota (0.43%), and Chytridiomycota (0.30%). At the class level, *Sordariomycetes* was the most abundant group, representing 60.2% of all sequences. Other prominent classes included Eurotiomycetes (9.8%), Agaricomycetes (9.8%), Pezizomycetes (3.7%), and Dothideomycetes (2.3%). Collectively, these five classes accounted for more than 85% of the classified fungal community at the class level (Figure S1), with 27% of ASVs not assigned beyond the Kingdom level. Taxa were further assigned into 64 fungal guilds across all samples. The fungal community was dominated by a few guilds, with the top three most abundant guilds being animal pathogen–endophyte–plant saprotrophs (28.3%), ectomycorrhizal fungi (24.2%), and undefined saprotrophs (17.9%). Less abundant guilds included animal parasites (4.3%), plant pathogens (2.2%), and other saprotrophic groups (Figure S2).

### 3.2 Plant Trait Variation

In the planted mesocosms containing marsh microbes (P+M+), *S. americanus* traits exhibited significant shifts in growth allocation and morphology with N input. Notably, R:S was significantly lower under high N input (*β* = −1.03, *p* = 0.006) across all plants. R:S also differed between age cohorts across both N treatments, with modern genotypes showing reduced belowground biomass allocation (relative to aboveground) than the ancestral genotypes (*β* = −0.830, *p* = 0.006). However, there was no significant N × age‐cohort interaction. In contrast, SLA (*β* = 0.60, *p* = 0.036) and SH (*β* = 0.37, *p* = 0.031) were both significantly higher under elevated N, reflecting increased aboveground growth under high N relative to low N.

### 3.3 Overall Influence of Plant Presence on Soil Fungal Communities (H1)

Consistent with H1, plant presence significantly increased fungal ASV diversity with mean fungal diversity higher in planted soils (i.e., rhizosphere soil) (mean = 6.33 ± 4.02) compared to unplanted soils (i.e., bulk soil) (mean = 5.58 ± 2.87) (GLMs: *F*_1,203_ = 13.83, *p* < 0.001; Table 1; Figure 3a). However, the magnitude of this effect depended on N level as a significant plant × N interaction was detected (*F*_1,203_ = 5.74, *p* = 0.017; Table 1, Figure 3a). As expected, marsh microbe treatment had a strong effect on fungal *ENSpie* diversity (*F*_1,203_ = 124.86, *p* < 0.001; Table 1, Figure 3a). *Post hoc* comparisons revealed that the effect of plant presence was only apparent under low N, where rhizosphere soils consistently exhibited higher *ENSpie* than bulk soils (6.31 ± 0.46 vs. 3.83 ± 0.32; respectively, *p* < 0.001; Table S1, Figure 3a). However, fungal diversity levels remained the same under high N regardless of the presence/absence of plants (*t* = −1.44, *p* = 0.151; Table S1, Figure 3a). Within rhizosphere soils, the effect of different N levels was apparent as *ENSpie* diversity was significantly higher under low N compared to high N conditions (*t* = 2.05, *p* = 0.042; Table S1, Figure 3a). However, no significant interaction among plant × N × marsh microbe was found (*F*_1,203_ = 0.007, *p* = 0.933; Table 1). These results were corroborated with the ‘plant effect’ analyses where fungal diversity increased in the presence of plants only under low N (Figure 3b) even when accounting for background microbial communities (Figure S3).

**TABLE 1:** Overall effects of plant presence, marsh microbe inoculation, and N input on fungal ASV (top panel) and guild (bottom) diversity (*ENSpie*).

Effect	df	Sum of squares	*F*	*p*
*ASV*				
Plant presence	1	3.766	13.826	< **0.001**
Marsh microbe	1	34.012	124.856	< **0.001**
Nitrogen input	1	0.089	0.327	0.568
Plant presence × Marsh microbe	1	1.046	3.839	0.051
Plant presence × Nitrogen input	1	1.564	5.742	**0.017**
Marsh microbe × Nitrogen input	1	0.014	0.051	0.822
Plant presence × Marsh microbe × Nitrogen input	1	0.002	0.007	0.933
Residuals	203	55.299	—	—
*Guild*				
Plant presence	1	0.011	0.101	0.751
Marsh microbe	1	5.392	48.216	**< 0.001**
Nitrogen input	1	0.226	2.019	0.157
Plant presence × Marsh microbe	1	0.208	1.858	0.174
Plant presence × Nitrogen input	1	0.021	0.189	0.664
Marsh microbe × Nitrogen input	1	0.094	0.836	0.362
Plant presence × Marsh microbe × Nitrogen input	1	0.092	0.823	0.366
Residuals	201	22.477	—	—

*Note:* Type II analysis of deviance from Gamma GLMs. Bold *p*‐values indicate *p* < 0.05.

Abbreviation: df, degrees of freedom.

**FIGURE 3:**
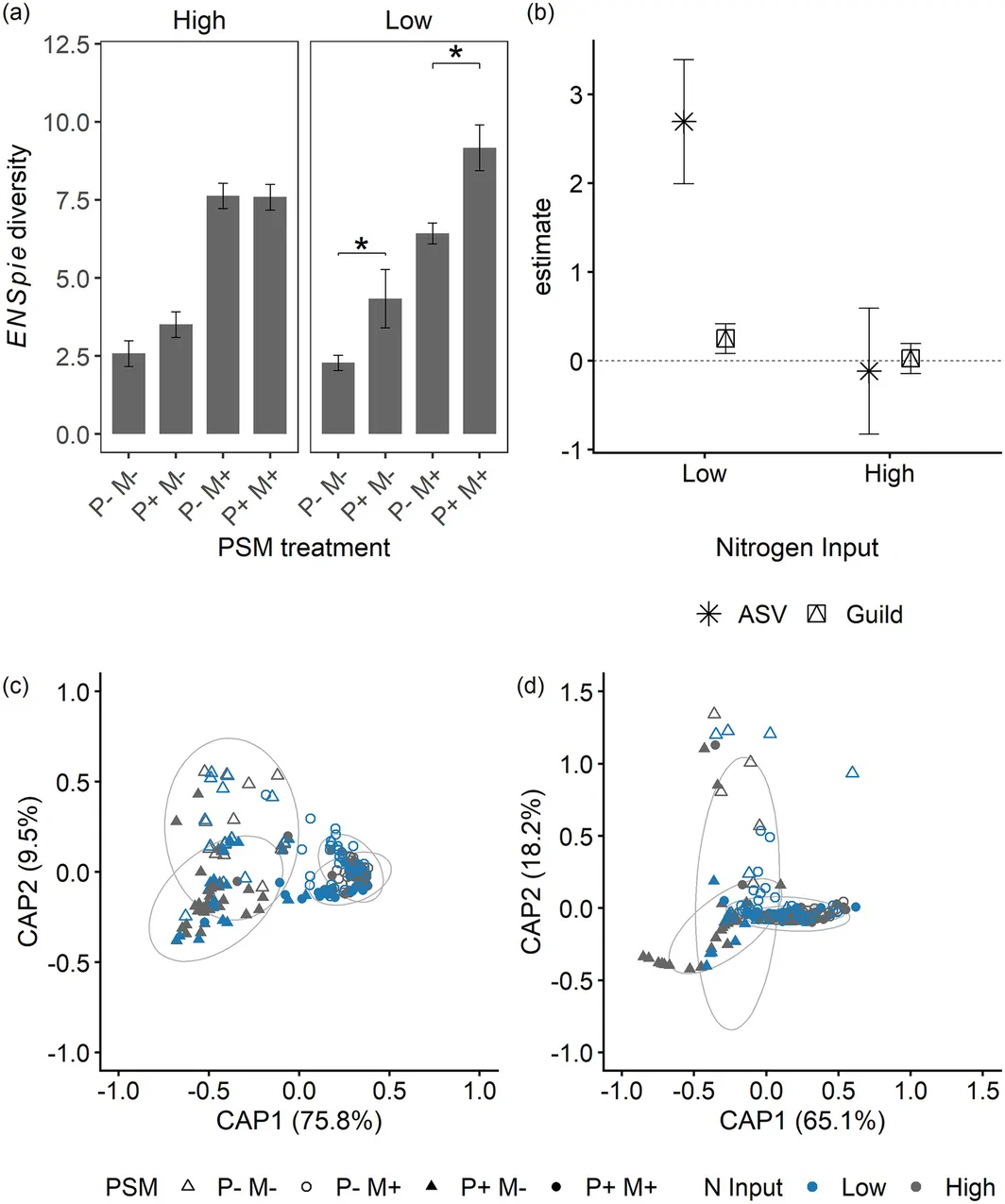
Influence of *S. americanus* presence on fungal communities across all samples (H1). (a) Raw mean ASV *ENSpie* diversity across the four PSM treatments under low and high N. Asterisks indicate significant differences (*p* < 0.05). (b) ‘Plant effect’ based on estimated marginal (EMMs) on *ENSpie* for ASV (star) and fungal guild (triangle) diversity under low and high N. Error bars are the standard error. db‐RDA of (c) ASV and (d) guild community composition constrained by PSM treatment and N input. Ellipses represent 95% confidence regions and axis percentages show constrained variance explained.

Fungal community compositions (*β*‐diversity) differed significantly among treatments (Table 2). PERMANOVA analyses revealed significant effects of plant presence (*F*_1,203_ = 5.72, *p* < 0.001; Table 2), N input (*F*_1,203_ = 2.38, *p* = 0.010; Table 2), and marsh microbe (*F*_1,203_ = 43.96, *p* < 0.001; Table 2). Significant interaction effects were detected for plant × marsh microbe (*F*_1,203_ = 3.59, *p* < 0.001; Table 2) and N × marsh microbe (*F*_1,203_ = 2.36, *p* = 0.008; Table 2), while the plant × N interaction and the three‐way interaction were not significant (both *p* > 0.18; Table 2). The PERMANOVA results were further supported by the db‐RDA, with a significant overall model (*F*_7,203_ = 7.920, *p* < 0.001; Figure 3c). The first two constrained axes were significant (CAP1: *F* = 42.02, *p* < 0.001; CAP2: *F* = 5.30, *p* < 0.001) and explained most of the constrained variation (Figure 3c). Forward model selection retained marsh microbe, plant presence, N input, and their interactions as significant predictors of fungal community structure.

**TABLE 2:** Permutational multivariate analysis of variance (PERMANOVA) of fungal community composition (across all samples) for ASV (top panel) and guild (bottom).

Effect	df	Sum of squares	*R* ^2^	*F*	*p*
*ASV*					
Marsh microbe	1	11.537	0.167	43.956	**< 0.001**
Plant presence	1	1.502	0.022	5.722	**< 0.001**
Plant presence × Marsh microbe	1	0.943	0.014	3.591	**< 0.001**
Nitrogen input	1	0.624	0.009	2.377	**0.010**
Nitrogen input × Marsh microbe	1	0.620	0.009	2.362	**0.008**
Plant presence × Nitrogen input	1	0.333	0.005	1.267	0.187
Plant presence × Nitrogen input × Marsh microbe	1	0.324	0.005	1.235	0.203
Residual	203	53.281	0.770	—	—
Total	210	69.163	1	—	—
*Guild*					
Marsh microbe	1	2.481	0.132	34.897	**< 0.001**
Plant presence	1	0.399	0.021	5.615	**< 0.001**
Plant presence × Marsh microbe	1	0.583	0.031	8.198	**< 0.001**
Nitrogen input	1	0.293	0.016	4.115	**0.004**
Nitrogen input × Marsh microbe	1	0.253	0.013	3.554	**0.012**
Plant presence × Nitrogen input	1	0.161	0.009	2.267	0.069
Plant presence × Nitrogen input × Marsh microbe	1	0.204	0.011	2.869	**0.028**
Residual	203	14.431	0.767	—	—
Total	210	18.805	1	—	—

*Note:* Bold *p*‐values indicate *p* < 0.05.

Abbreviation: df, degrees of freedom.

Guild level analyses revealed different patterns from ASV analyses. Across all samples, guild diversity showed limited response to plant presence (mean guild diversity rhizosphere = 1.82 ± 0.657; bulk soil = 1.86 ± 0.691; *F*_1,201_ = 0.10, *p* = 0.751; Table 1; Figure 3b) and N input (*F*_1,201_ = 2.02, *p* = 0.157; Table 1). Marsh microbe inoculation, however, significantly influenced guild *ENSpie* diversity (*F*_1,201_ = 48.22, *p* < 0.001; Table 1). However, the PERMANOVA revealed that differences in the fungal community guild compositions (*β*‐diversity) were influenced by plant presence (*F*_1,203_ = 5.62, *p* < 0.001; Table 2), N input (*F*_1,203_ = 4.12, *p* = 0.004; Table 2), and marsh microbe (*F*_1,203_ = 34.90, *p* < 0.001; Table 2). Individual effects of these factors depended on each other as significant interactions were also detected for plant × marsh microbe (*F*_1,203_ = 8.20, *p* < 0.001), N × marsh microbe (*F*_1,203_ = 3.55, *p* = 0.012), and the three‐way interaction among plant presence, N input, and marsh microbe inoculation (*F*_1,203_ = 2.87, *p* = 0.028) (Table 2). These findings were supported by db‐RDA analyses which showed a significant overall model (*F*_7,203_ = 7.52, *p* < 0.001), explaining 20.6% of total variation in guild composition and significant constrained axes (Figure 3d). Additional information is presented in Supporting Information S1.

### 3.4 Influence of Intraspecific *S. americanus* Trait Variations on Rhizosphere Fungi Across Age Cohorts (H2)

We found partial support for H2 where the influence of heritable trait variation on fungal ASV diversity was limited and context dependent. Multimodel inference analyses indicated that the biggest difference in rhizosphere fungal *ENSpie* diversity was found between the marsh microbe mesocosms and no‐microbe control mesocosms (*β* = 0.65 ± 0.12, *p* < 0.001; Table 3), regardless of N treatment. N treatment showed a weak but significant effect on fungal diversity, with diversity lower under high N compared to low N conditions (*β* = −0.26 ± 0.12, *p* = 0.029; Table 3). SDi, SN, R:S, BG, and SLA all had low relative importance (Table 3) on shaping fungal diversity (Σ*w* ≤ 0.44; Table 3). Fungal ASV diversity also did not significantly vary according to age cohort (Σ*w* = 0.27; *β* = −0.11 ± 0.11, *p* = 0.32; Table 3). Given the strong effect of marsh microbe inoculation treatment, we also analysed data using only the P+M+ (i.e., mesocosms containing both plant and marsh microbes). Similar patterns were observed (see Supporting Information S1).

**TABLE 3:** Model‐averaged coefficients and predictor importance for rhizosphere fungal *ENSpie* diversity responses (P+ samples) at ASV (top panel) and guild (bottom) levels.

Predictor	Estimate (*β*)	Adjusted SE	*z*	*p*	Relative importance (Σ*w*)	Model count
*ASV*						
(Intercept)	1.599	0.112	14.253	**< 0.001**	—	—
Marsh microbe	0.646	0.119	5.447	**< 0.001**	1	95
Nitrogen input	−0.257	0.118	2.179	**0.029**	1	95
IW	0.088	0.053	1.644	0.100	0.570	54
SDi	−0.077	0.054	1.429	0.153	0.440	43
SLA	0.063	0.051	1.215	0.224	0.360	37
SN	−0.062	0.058	1.062	0.288	0.280	30
Age cohort	−0.113	0.113	1.000	0.317	0.270	34
Marsh microbe × Nitrogen input	0.137	0.197	0.694	0.488	0.200	26
R:S	0.038	0.063	0.610	0.542	0.190	25
BG	−0.019	0.062	0.312	0.755	0.160	22
*Guild*						
(Intercept)	0.422	0.070	6.037	**< 0.001**	—	—
Marsh microbe	0.361	0.063	5.711	**< 0.001**	1	34
Nitrogen input	0.025	0.062	0.402	0.688	1	34
Age cohort	−0.164	0.067	2.445	**0.015**	0.860	28
SDi	−0.045	0.031	1.431	0.153	0.520	20
IW	0.027	0.030	0.905	0.366	0.280	13
BG	0.026	0.032	0.806	0.421	0.190	8
SN	0.023	0.037	0.614	0.539	0.160	7
Marsh microbe × Nitrogen input	0.073	0.118	0.622	0.534	0.140	6
SLA	0.016	0.030	0.519	0.604	0.130	6
R:S	−0.013	0.030	0.425	0.671	0.100	4

*Note:* Estimates are derived from multimodel inference using the ΔAICc ≤ 4 candidate model set. Relative importance (Σ*w*) reflects the summed Akaike weights across all models including the predictor. Model count indicates the number of top models in which each predictor was retained. Bold *p*‐values indicate *p* < 0.05.

Rhizosphere soil fungal community composition significantly varied according to *S. americanus* age cohort, marsh microbe, and N treatments. PERMANOVA analyses revealed that, overall, fungal ASV communities responded more strongly to abiotic factors than to variation in heritable plant traits (Figure 4c; Table S2). N treatment significantly influenced fungal community composition depending on marsh microbe inoculation as a significant interaction was found between PSM treatment and N input (*R* ^2^ = 0.014, *F*_1,94_ = 1.87, *p* = 0.039; Figure 4c; Table S2). Fungal community compositions also differed between ancestral and modern genotypes (*R* ^2^ = 0.014, *F*_1,94_ = 1.83, *p* = 0.047; Figure 4a,c; Table S2). This was partly reflected in the specific ASVs that were strongly associated with either ancestral or modern plants based on indicator species analyses. For example, two and 16 ASVs were significantly associated with ancestral and modern cohorts, respectively. Among these, ASV_2136 (*Ascomycota*; *Pezizomycetes*; *Pezizales* sp.) was uniquely associated with ancestral plants, whereas ASV_1358 (*Ascomycota*; *Sordariomycetes*; *Zopfiella* sp.) was strongly associated with modern plants. We further identified specific ASVs that were strongly associated with plant age cohort under contrasting N conditions (Table S3).

**FIGURE 4:**
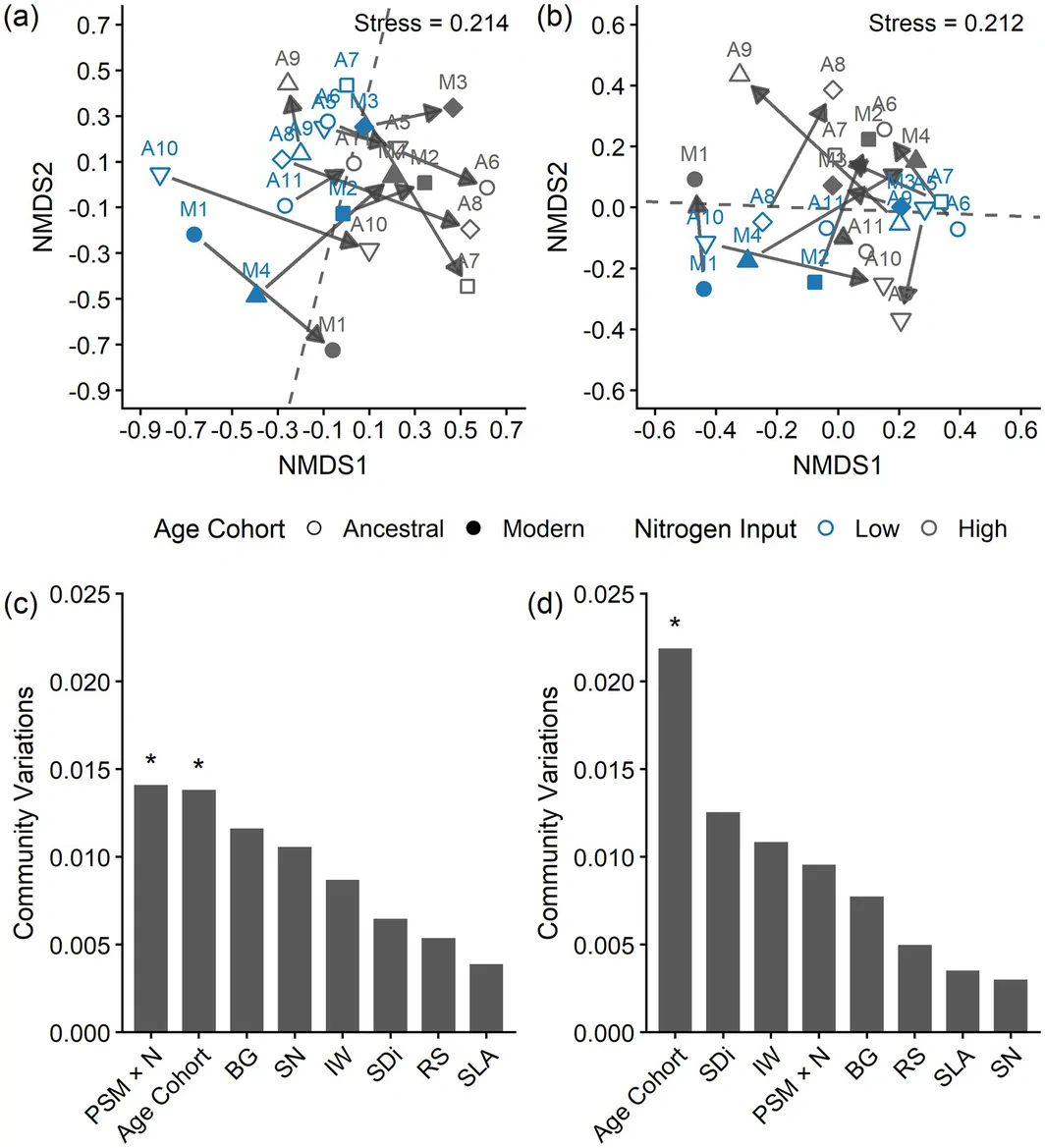
Non‐metric multidimensional scaling (NMDS) ordination of rhizosphere fungal community composition based on Bray–Curtis dissimilarity for (a) ASV and (b) guild data for P+ (H2). Each point/symbol represents the mean community profile (abundances averaged across replicates) for a plant genotype under an N treatment and distinguished by plant age cohort (ancestral = open symbols; modern = filled symbols). Arrows indicate the directional shift from low to high N for each genotype. Dashed line indicates arbitrary separation between N treatments. PERMANOVA *R* ^2^ results are shown for ASV (c) and guild (d). Bars represent marginal *R* ^2^ values for each factor, with asterisks for *p* < 0.05.

db‐RDA results were consistent with the PERMANOVA results. Constrained predictors collectively explained a small but significant portion of ASV rhizosphere community variation (*R* ^2^ = 0.024; adj. *R* ^2^ = 0.009), with both N input (*F*_1,101_ = 1.58, *p* = 0.027) and plant age cohort (*F*_1,101_ = 1.54, *p* = 0.032) contributing significantly to variation in fungal ASV composition. Among canonical axes, only CAP1 was significant (*F*_1,101_ = 1.85, *p* = 0.029), whereas CAP2 was not (*p* = 0.147).

SEM analyses identified limited direct and indirect (i.e., via plant traits) effects of N on fungal diversity (Figure 2). Models showed good overall fit (*χ* ^2^ = 5.23, *p* = 0.156; Fisher's *C* = 8.15, *p* = 0.227), explaining a modest proportion of variation in *ENSpie* diversity (*R* ^2^ = 0.41). N treatment showed a significant direct impact on ASV *ENSpie* diversity (estimate = −0.236, *p* = 0.041; Table S5, Figure 2), as did marsh microbe inoculation (estimate = 0.673, *p* < 0.001; Table S5, Figure 2). Plant age cohort had limited influence on *ENSpie* diversity (*p* = 0.376; Table S5, Figure 2). While N treatment influenced R:S (*β* = −0.335, *p* < 0.001; Table S5, Figure 2), R:S had no effect on fungal diversity (Table S5, Figure 2). Furthermore, R:S and BG were significantly lower among modern than ancestral plants (R:S: *β* = −0.223, *p* = 0.018; BG: *β* = −0.193, *p* = 0.041; Table S5, Figure 2). However, variations in these two heritable traits between age cohorts did not extend to fungal diversity (Table S5, Figure 2), nor did any of the other plant traits. IW was significantly correlated with BG (*β* = 0.247, *p* = 0.009) and SN (standardized *β* = 0.208, *p* = 0.0334) (Table S5, Figure 2).

Guild‐level analyses showed slightly different patterns found at the ASV level. Guild *ENSpie* diversity was not significantly correlated with any of *S. americanus* traits, nor did it differ according to N input (*β* = 0.03 ± 0.06, *p* = 0.688; Table 3) based on the multimodel analyses. Across all P+ samples, guild diversity showed strong differences between the microbial control group and those receiving marsh microbes (Σ*w* = 1.00, *β* = 0.36 ± 0.06, *p* < 0.001; Table 3). In contrast to ASV‐level results, guild diversity differed significantly between age cohorts, with lower diversity observed in modern relative to ancestral plants (Age: *β* = −0.16 ± 0.07, *p* = 0.015, Σ*w* = 0.86; Table 3). The guild compositions of rhizosphere soil communities also did not differ across all treatments except for between age cohorts (*F*_1,94_ = 3.06, *p* = 0.019; Table S2). Partial db‐RDA conditioning further indicated small but significant effects of N input and stem diameter, collectively explaining 5.55% of variation in guild composition (adj. *R* ^2^ = 0.041).

Indicator species analyses showed strong associations of specific guilds with N, age cohort, and genotype combinations. For example, six guilds were significantly associated with low N conditions, including plant–wood saprotrophs, while only two guilds (e.g., dung saprotrophs) were linked to high N (Table S4a). We also detected guilds that were strongly linked to age cohort (Table S4b), genotype (Table S4c), and age cohort × N (Table S4d). For example, three guilds (wood saprotrophs, parasite–saprotrophs, and mixed pathogen–saprotrophs) were strongly associated with ancestral plants under low N, whereas a specialized bryophyte parasite/plant saprotroph/wood saprotroph guild complex was enriched with modern plants under high N (Table S4d).

SEM analyses at the guild level demonstrated an acceptable overall fit (*χ* ^2^ = 5.23, *p* = 0.156; Fisher's *C* = 8.15, *p* = 0.227) but explained relatively low variation in *ENSpie* guild diversity (Table S5). N treatment showed no direct effect on guild *ENSpie* diversity (estimate = 0.021, *p* = 0.730; Table S5), whereas marsh microbe treatment exerted a strong positive influence (*β* = 0.369, *p* < 0.001; Table S5). In contrast, plant age cohort had a significant negative direct effect on guild *ENSpie* diversity (estimate = −0.189, *p* = 0.004; Table S5).

Corresponding analyses using Shannon diversity yielded qualitatively similar patterns and are reported in Supporting Information S2 (Figures S4 and S5; Tables S6–S9).

### 3.5 Differences in Rhizosphere Soil Fungal Communities Reflect G × E (H3)

We found evidence of genotype × environment (G × E) interactions, where associated fungal diversity (or *S. americanus* association with fungal communities, *sensu* the extended phenotype hypothesis) differed according to genotype in response to N treatment. BLUPs estimates revealed genotype‐specific N responses that varied in both magnitude and direction depending on the presence of marsh microbes (Figure 5a). For example, ancestral genotypes A11 and A5 showed contrasting patterns in their estimated mean fungal diversity trait value when comparing low and high nitrogen treatments in the presence of marsh microbe (A11: 0.714 vs. 0.051; A5: −0.154 vs. 0.339; Figure 5a). Notably, a range of genotypic variation was observed as patterns of G × E differed between plants receiving the marsh microbe inoculation (P+M+) and plants in the marsh microbe control group (P+M−) (Figure 5a). Genotypic variation in rhizosphere fungal diversity associated with plants that received the marsh microbe inoculation showed a greater range of variation than in the P+M− in response to N treatments (Figure 5a). In contrast, fungal guild diversity showed a different pattern, with weaker genotype‐specific N responses in the presence of marsh microbes and relatively stronger and more consistent responses in the microbe control group (Figure 5b).

**FIGURE 5:**
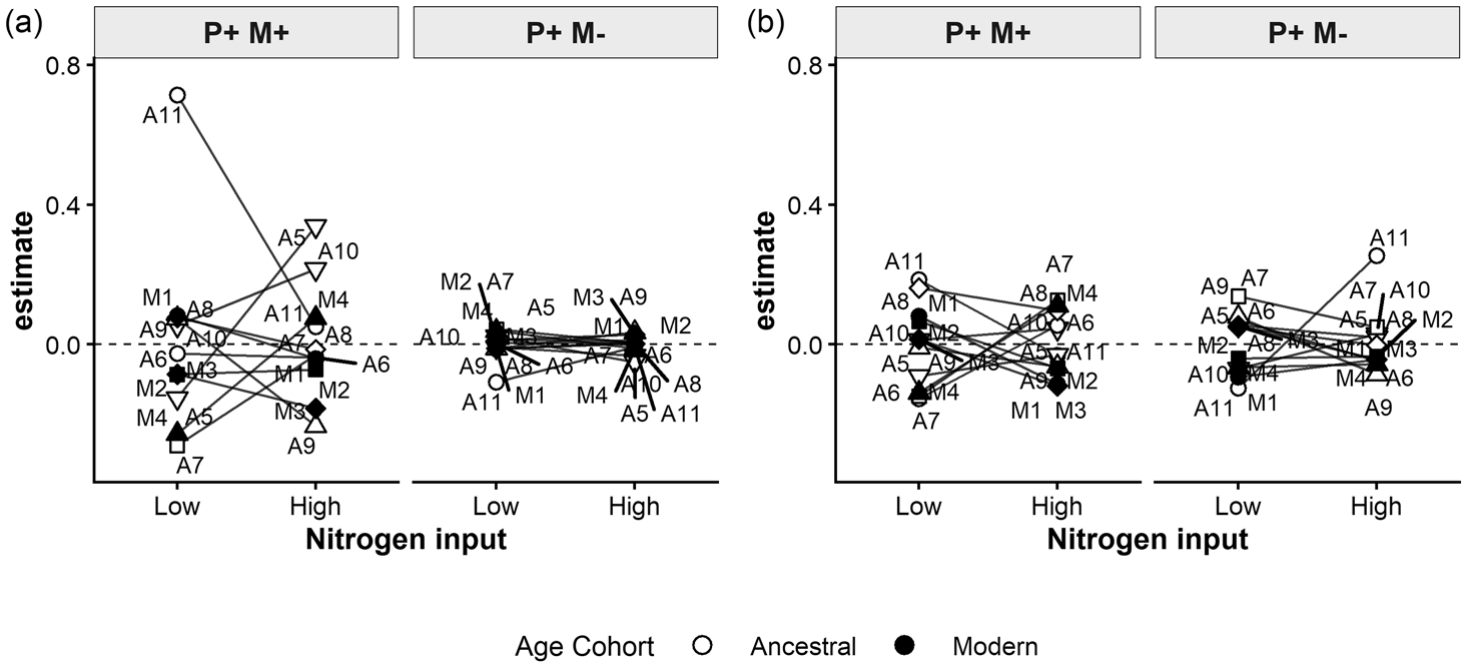
Differences among *S. americanus* genotypes in associated rhizosphere fungal diversity in response to N and marsh microbe inoculation. Best linear unbiased predictor (BLUP) estimation (similar to a reaction norm) of (a) ASV and (b) Guild *ENSpie* diversity in the presence of marsh microbes (P+M+) and control conditions (P+M−) under low and high N. Filled symbols indicate modern genotypes and open symbols represent ancestral genotypes.

## 4 Discussion

Our findings offer further evidence that rhizosphere microbial communities are subject to the influence of associated plants (Des Roches et al. 2018; Lumibao et al. 2020), but also show that the magnitude of influence depends in part on the prevailing environmental conditions. Our work also extends understanding of plant‐microbe relationships by showing that associations are dynamic over time (i.e., across plant age cohorts). First, we found that the presence of *S. americanus* significantly altered the taxonomic and functional diversity and composition of rhizosphere fungal communities, where the magnitude of influence was contingent on N exposure. Under low N, plant presence increased fungal taxonomic (ASV) diversity. The presence of plants exerted less influence under high N conditions where no significant difference in diversity and composition between rhizosphere and unplanted bulk soils was detected. We found limited support for our hypothesis that heritable plant trait variation strongly influences rhizosphere fungal diversity and composition. However, we detected evidence for G × E interactions including variation in plasticity among genotypes in their associations with rhizosphere fungal communities in response to N conditions. Furthermore, we found evidence that fungal associations can shift over time, where ancestral and modern genotypes exhibited different fungal associations regardless of N conditions. These findings indicate that plant‐fungal associations can be dynamic (i.e., can shift over time) and that high N reshapes relationships by reducing variability in plant‐fungal interactions.

### 4.1 Influence of Plant Presence on Marsh Soil Fungi Depends on N Level

To determine the magnitude of *S. americanus* influence on soil fungi, we first explored differences in planted (i.e., rhizosphere) vs. unplanted (i.e., bulk) soils (H1). Our findings provide strong support for H1, demonstrating that rhizosphere fungal community diversity and composition are distinct from bulk soil, though this pattern was more apparent under low N conditions. Under low N, the presence of *S. americanus* increased taxonomic (ASV) *α*‐diversity of fungi without a concomitant change in guild diversity. This may indicate that *S. americanus* recruits fungal taxa into the rhizosphere zone that are likely to belong to same fungal guilds or perform similar ecological roles (e.g., saprobes) (Louca et al. 2018). The increase in taxonomic diversity was accompanied by shifts in ASV composition. Though guild diversity did not increase, guild composition differed between rhizosphere and bulk soils, suggesting that the presence of *S. americanus* led to distinct fungal guilds (e.g., saprotrophs and mutualists) in rhizosphere soils compared to bulk soils. It is likely that under low N, *S. americanus* provides carbon‐rich substrates and modifies N availability through root exudates and altered redox conditions, supporting a richer and compositionally distinct fungal community in rhizosphere than bulk soils (Broeckling et al. 2008; Philippot et al. 2013).

The influence of *S. americanus* on fungal communities was diminished under high N, where fungal diversity did not differ between rhizosphere and bulk soils. It is possible that fungi might be responding directly to high N, with N acting as a strong pressure that promotes the growth of specific N‐dependent fungal taxa. High N in the soil could alter the amount and quality of rhizodeposition and favour fungal taxa with higher nutrient affinity, thereby weakening the plant‐mediated niche differentiation that otherwise influences rhizosphere fungal communities (Kuzyakov and Xu 2013; Moreau et al. 2019). This finding might also indicate that when N is readily available, fungi are less reliant on plant‐derived resources, diminishing the influence of plants over fungi and, in some cases, reducing fungal diversity by promoting select species that dominate under nutrient‐rich conditions. Notably, other studies have demonstrated that nutrient enrichment can shift fungal assemblage composition depending on the type of N added (Garces et al. 2026) through an increase in fungal abundance and the proliferation of putative fungal denitrifiers depending on the type of N (Kearns et al. 2019).

### 4.2 Differences in Rhizosphere Soil Fungal Communities Reflect G × E but Not Heritable Plant Trait Variation in Response to N

Prior work, including our own (Lumibao et al. 2020), has shown that intraspecific plant trait variation can shape rhizosphere microbial communities. We detected significant differences in several *S. americanus* traits in response to N treatment (e.g., R:S decreased under high N), corroborating findings from prior work on *S. americanus* (e.g., Lumibao et al. 2022; Vahsen, Blum, et al. 2023; Vahsen, Kleiner, et al. 2023). The observed plant trait differences did not, however, extend to the associated fungal communities as we found no direct influence of plant traits on the diversity and composition of fungal communities. Furthermore, while fungal communities responded directly to differences in N level, their responses were also not mediated by variation in plant traits (i.e., fungal responses to N were not indirectly influenced by specific plant traits) according to SEM analyses.

Our findings indicate that plant‐associated rhizosphere fungal communities reflect genotype × environment (G × E) interactions (i.e., variable reaction norms). We detected G × E differences in rhizosphere fungal diversity that reflect variation among genotypes in their associations with fungi under different N conditions. Notably, signatures of variation differed between ancestral and modern genotypes, further indicating that plant‐fungal associations can be plastic, and that plasticity differs among genotypes. These results are consistent with other findings showing that environmental stress can influence plant trait expression and associated microbial responses (Lumibao et al. 2022; Nicotra et al. 2010; Garces et al. 2026; Walker and Emery 2026). In general, variation in fungal communities was lower under high N, consistent with theoretical predictions that only a few taxa or associations might become more dominant when stress is elevated (Bertness and Callaway 1994). Greater genotypic variation was observed when considering fungal taxonomic diversity compared to guild diversity. Consistent with findings from other studies, this might indicate that taxonomic diversity may shift because of differences in recruitment and competitive sorting among fungal taxa, whereas broader guild structure may remain relatively stable (Moreira et al. 2025; Zhou et al. 2026).

Variation in reaction norms was also greater when marsh microbes were present compared with the control group, suggesting that genotype effects on rhizosphere fungal communities become more apparent when a diverse inoculum pool is available for recruitment and filtering rather than when species pools are reduced in soils. These results also suggest that rhizosphere communities are heritable and thus represent an extended root phenotypic trait of plants (i.e., *sensu* the extended phenotype hypothesis) (de la Fuente Cantó et al. 2020). Similar G × E interactions have been reported in other dominant salt marsh plant species (e.g., Hanley et al. 2025).

Evidence of greater influence from genotypic versus trait variation on fungal communities contrasts with findings of other studies demonstrating that fungal communities are responsive to intraspecific plant trait variation, including work done on the marsh grasses *Spartina alterniflora* and *Phragmites australis* (Lumibao et al. 2020; Hanley et al. 2025). Our prior work on *S. americanus* also detected weak but positive associations between endophytic fungi and intraspecific trait variation (Lumibao et al. 2022). These discrepancies might be a consequence of our study design—e.g., a wider range of associated phenotypic trait variation might have been captured had we examined different populations or additional genotypes and/or had we measured other traits of *S. americanus* —but other studies have illustrated that abiotic filtering can sometimes outweigh host control of microbial assembly (Alzarhani et al. 2019). In our study, high N reduced differences in fungal communities driven by plant traits.

It might also be possible that the microbiota in the marsh soil inoculum used in this study were not sensitive to the influence of *S. americanus* since the inoculum was not derived from the plants' native rhizosphere (i.e., the inoculum was not obtained from the Chesapeake Bay marsh where we sourced *S. americanus* seeds). Had we used native inoculum, we might have captured a greater response of fungal communities to heritable trait variation in *S. americanus* .

### 4.3 Ancestral and Modern Genotypes Differ in Extent of Fungal Associations

We found support for distinct fungal communities between ancestral and modern *S. americanus* genotypes regardless of N condition, suggesting that fungal recruitment is a dynamic process that can vary over time (or at least across plant generations). The composition of rhizosphere fungal communities associated with ancestral and modern *S. americanus* genotypes was categorically distinct, particularly at the guild level, with modern genotypes having lower fungal guild (but not ASV) *α*‐diversity and a greater number of strongly associated fungal taxa (i.e., indicator taxa) regardless of N. These results indicate that fungal associations can change over time (in this instance, across plant generations). These findings are consistent with other resurrection studies showing that ancestral and modern *S. americanus* differ in responses to environmental stress over time (Lumibao et al. 2022; Vahsen, Blum, et al. 2023; Vahsen, Kleiner, et al. 2023). Our findings might in part, however, reflect responses of fungal associates to changing environmental pressures. Thus, depending on the rate of responses of both fungi and plants, plant‐fungal interactions might be a dynamic outcome of separate and combined responses. These results build on evidence from prior studies of *S. americanus* evolution across a century (Blum et al. 2021; Vahsen, Blum, et al. 2023) and that microbial mediation of plant stress tolerance can vary with plant genotype (Lumibao et al. 2022). Notably, rapid evolutionary changes in *S. americanus* extend to belowground structures, which can alter vital ecosystem properties (Vahsen, Blum, et al. 2023; Vahsen, Kleiner, et al. 2023). Our findings suggest that changes can extend to belowground ecological interactions.

### 4.4 Conclusion

Our study suggests that plant evolution and ecological interactions between plants and fungi might be important factors shaping the persistence of coastal ecosystems under anthropogenic pressures like nutrient enrichment. As *S. americanus* is a dominant species and ecosystem engineer in coastal marshes, shifts in fungal associates could alter vital coastal ecosystem processes. For example, changes in fungal community composition may influence organic matter decomposition and N transformation as salt marsh fungi contribute to plant detritus breakdown and participate in denitrification processes (Kearns et al. 2019; Starr et al. 2022). Our results also demonstrated that plant‐fungal associations are dynamic (i.e., change over time), and the extent to which plants influence associated fungal communities can be weakened by a strong environmental disturbance. Understanding functional linkages between plants and microbes, how they shift under environmental change, and how functional shifts shape ecosystem processes, could improve efforts to predict the near‐term future and long‐term fate of at‐risk coastal marsh ecosystems.

## Author Contributions

C.Y.L. and M.J.B. conceived and designed the experiment; Y.L. conducted the greenhouse experiment, with assistance from C.Y.L.; Y.L. performed trait measurement and microbial work; Y.L. conducted the analysis with assistance from C.Y.L.; Y.L. and C.Y.L. wrote the first draft of the manuscript; Y.L., C.Y.L., and M.J.B. contributed to the final manuscript.

## Funding

This work was supported by the Texas A&M University—Corpus Christi (TAMU‐CC) Texas Comprehensive Research Fund (TCRF) awarded to C.Y.L. and the TAMU‐CC Student Research Grant to Y.L.

## Disclosure

*Benefit‐Sharing Statement*: Benefits Generated: Benefits from this research accrue from the sharing of our data and results on public databases as described above.

## Ethics Statement

The authors have nothing to report.

## Conflicts of Interest

The authors declare no conflicts of interest.

## Supporting information


**Figure S1:** Mean relative abundances of fungal phyla (a, b) and classes (c, d) across PSM treatments (P+M+, P−M+, P+M−, P−M−) under nitrogen inputs (N−: Low; N+: High) and plant age cohorts (Ancestral, Modern). Ancestral and modern plant cohorts are shown separately from unplanted soils. Relative abundance was calculated for each sample and averaged across samples within each treatment group. Only the top taxa are shown, with remaining taxa grouped as “Other.”
**Figure S2:** Mean relative abundances of fungal ecological guilds under nitrogen treatments (Low and High) across different PSM treatments (P+M+, P−M+, P+M−, P−M−) for ancestral and modern plant cohorts (a), and unplanted soils (b). Relative abundance was calculated for each sample and averaged across samples within each treatment group. Only the top 10 most abundant guilds are shown, with remaining guilds grouped as “Other.”
**Figure S3:** Estimated marginal means (±SE) of *ENSpie* showing the stepwise plant effect across nitrogen treatments (Low and High). Plant effects were calculated using the stepwise difference approach described in the Methods, where fungal diversity of plants with marsh microbes (P+M+) was first contrasted with plant controls lacking marsh microbes (P+M−) (Diff 1), and fungal diversity of soils with microbes but no plants (P−M+) was contrasted with microbe controls (P−M−) (Diff 2) within each nitrogen treatment and replicate. The final plant effect was obtained by subtracting Diff 2 from Diff 1, thereby accounting for background microbial communities independent of plants. Circles represent ASV‐based estimates and triangles represent guild‐based estimates. The horizontal dashed line indicates zero, corresponding to no net plant effect after background subtraction.
**Table S1:** Pairwise comparisons of estimated marginal means (EMMs) for *ENSpie* across plant presence and nitrogen input (H1). Values are Tukey‐adjusted pairwise comparisons of back‐transformed estimated marginal means averaged across marsh microbe levels. Bold *p*‐values indicate *p* < 0.05; df = degrees of freedom.
**Table S2:** PERMANOVA results testing the effects of plant traits, plant age cohort, and marsh microbe and nitrogen treatment on fungal community composition at the ASV (top) and guild (bottom) levels. Significant effects in bold (*p < 0.05); df = degrees of freedom.
**Table S3:** Indicator ASVs identified by multilevel pattern analysis (IndVal.g). Indicator species analysis was performed using multipatt (package indicspecies) with the IndVal.g association function. ASVs were tested for significant association with the nitrogen Input × plant age cohort combined factor. Significance was assessed using 9999 permutations (*α* = 0.05).
**Table S4:** Indicator ecological guilds identified by multilevel pattern analysis (IndVal.g). Indicator guild analysis was conducted using multipatt (indicspecies) with the IndVal.g association function. Guilds were tested for significant association with (a) nitrogen input (Low vs. High), (b) plant age cohort (Ancestral vs. Modern), (c) plant genotype, and (d) the nitrogen input × plant age cohort combined factor. Significance was assessed using 9999 permutations (*α* = 0.05).
**Table S5:** Structural equation model (SEM) results for fungal diversity (*ENSpie*). Standardized SEM coefficients are shown for ASV‐based (top panel) and ecological guild‐based (bottom) datasets. Predictors include plant traits and experimental factors: marsh microbe, nitrogen input, and plant age cohort. Values reported are unstandardized estimates, standard errors (SE), test statistics (*z*), *p* values, and standardized coefficients (Std. Estimate). Bold *p*‐values indicate *p* < 0.05.

**Figure S4:** Raw ASV‐level Shannon (mean ± SE) across plant–soil‐microbe (PSM) treatments under low and high N. Treatments were combinations of plant presence (P+/P−) and marsh microbe inoculation (M+/M−).
**Figure S5:** Piecewise structural equation model (SEM) describing factors influencing ASV‐level rhizosphere Shannon diversity. The model quantifies direct and indirect effects of marsh microbe inoculation, N input, plant age cohort, and plant heritable traits on fungal diversity. Arrows represent significant causal paths (*p* < 0.05), and numbers indicate standardized path coefficients. Only significant relationships are depicted.
**Table S6:** Overall effects of plant presence, marsh microbe inoculation, and N Input on fungal ASV (top panel) and guild (bottom) diversity (Shannon) (H1). Type II analysis of deviance from Gamma GLMs (log link). Bold *p*‐values indicate *p* < 0.05; df = degrees of freedom.
**Table S7:** Pairwise comparisons of estimated marginal means (EMMs) for Shannon across plant presence and marsh microbe inoculation conditions. Results are based on Gaussian GLMs (identity link). Values are Tukey‐adjusted pairwise comparisons averaged across N input levels. Bold *p*‐values indicate *p* < 0.05; df = degrees of freedom.
**Table S8:** Model‐averaged coefficients and predictor importance for rhizosphere fungal Shannon diversity responses (only P+ samples) at ASV (top panel) and guild (bottom) levels (H2). Estimates are derived from multimodel inference using the ΔAICc ≤ 4 candidate model set. Relative importance (Σ*w*) reflects the summed Akaike weights across all models including the predictor. Model count indicates the number of top models in which each predictor was retained. Bold *p*‐values indicate *p* < 0.05.
**Table S9:** Structural equation model (SEM) results for fungal diversity (Shannon). Standardized SEM coefficients are shown for ASV‐based (top panel) and ecological guild‐based (bottom) datasets. Predictors include plant traits and experimental factors: marsh microbe inoculation, N input, and plant age cohort. Values reported are unstandardized estimates, standard errors (SE), test statistics (*z*), *p* values, and standardized coefficients (Std. Estimate). Bold *p*‐values indicate *p* < 0.05.

## Data Availability

Upon acceptance of the manuscript, raw Illumina sequence data will be deposited in the NCBI Sequence Read Archive (SRA) under BioProject PRJNA1531213. All input files and metadata used for analyses are available at Lumibao Lab Github (https://github.com/LumibaoLab).
