# Supplementary material for: Rhizosphere Fungal Community Responses to Nitrogen Addition Reflect Genotypic Versus Trait Variation in the Marsh Sedge Schoenoplectus americanus Over Time
Source: Mol Ecol. 2026 Oct 14;35(20):e70588. doi: 10.1111/mec.70588 (PMC13667335; doi:10.1111/mec.70588)
Supplement: Supplementary file 1 [file MEC-35-e70588-s001.docx]

**Supporting Information 1 for:**

**Rhizosphere fungal community responses to elevated nitrogen reflect genotypic versus trait variation in the marsh sedge *Schoenoplectus americanus* over time**

Yue Liu^1^, Michael J. Blum^2^ and Candice Y. Lumibao^1^

^1^Department of Life Sciences, Texas A&M University – Corpus Christi, Corpus Christi, Texas, USA

^2^Department of Ecology & Evolution, University of Tennessee – Knoxville, Knoxville, Tennessee, USA

**Table of Contents:**

| **Methods** | Page 2 |
| --- | --- |
| **References** | Page 5 |
| **Results** | Page 6 |
| **Figures S1** | Page 8 |
| **Figures S2** | Page 9 |
| **Figures S3** | Page 10 |
| **Tables S1** | Page 11 |
| **Tables S2** | Page 12 |
| **Tables S3** | Page 13 |
| **Tables S4** | Page 16 |
| **Tables S5** | Page 17 |
|  |  |

**METHODS**

*Study Site and Species Background*

Corn Island is a brackish tidal marsh located within the Rhode River estuary, which is a sub-estuary of Chesapeake Bay in Maryland, USA. Vegetation in the marsh is composed of *Schoenoplectus americanus*, *Spartina patens*, *Distichlis spicata*, and, in some areas, *Phragmites australis*. The stratigraphic layers were previously dated using radioisotope techniques (210Pb and 137Cs), and seeds recovered from these layers have been successfully germinated to grow ancestral and descendant genotypes (Vahsen, Blum, et al., 2023; Vahsen, Kleiner, et al., 2023).

*Schoenoplectus americanus* (formerly *Scirpus americanus*) is an ecologically dominant C₃ perennial sedge native to brackish and freshwater marshes along the Atlantic and Gulf Coasts of North America. It typically grows 1–2 meters tall and reproduces both sexually through prolific annual seed production and asexually through rhizome growth (USDA, 2023), forming dense stands that contribute to carbon sequestration and resistance to erosion. *S. americanus* plays an important role in stabilizing marsh sediments, modulating hydrology, and supporting nutrient cycling through its high productivity and dense belowground biomass (Barbier et al., 2011; Zedler & Kercher, 2005).

*Soil Substrate Composition and Preparation*

Each pot was filled with a total of 3.37 kg of soil, composed of 5% live marsh soil and 95% sterilized background soil. The background soil consisted of a 1:1 (v/v) mixture of topsoil (Black Velvet® Premium Top Soil, 40 lb) and sand (QUIKRETE® Premium Play Sand, 50 lb). Marsh soil was collected from Oso Bay marsh near Corpus Christi (TX, USA), sieved to remove debris, and stored on ice before use. Soil mixing and transplanting were conducted in a designated greenhouse space located at the Texas AgriLife Research Mariculture Laboratory in Flour Bluff, TX.

*Molecular work*

Fungal ITS amplicons were generated using a two-step PCR approach (Van Bael & Arnold) with Illumina adapter-tagged primers targeting the fungal ITS region. The gene-specific portions correspond to ITS1f and ITS2 fungal primers previously validated for fungal community analyses (e.g., Lumibao et al., 2018). Primer sequences (5′–3′) are listed below.

Forward primer: ITS1f_adap: CACTCTTTCCCTACACGACGCTCTTCCGATCTCTTGGTCATTTAGAGGAAGTAA

Reverse primer: ITS2_adap: GTGACTGGAGTTCAGACGTGTGCTCTTCCGATCTGCTGCGTTCTTCATCGATGC

For first-round amplification, reactions were prepared in 15 µL volumes using Phusion Flash High-Fidelity Master Mix (ThermoFisher F548). Each reaction contained 6.00 µL Phusion Flash Master Mix, 0.20 µL each of forward (ITS1f_adap) and reverse (ITS2_adap) primers (10 µM), 0.75 µL bovine serum albumin (BSA; 20 mg mL⁻¹, NEB), 5.85 µL DNase-free water, and 2.00 µL template DNA (~20 ng). The PCR conditions (run in triplicate reactions for each sample) were as follows: initial denaturation at 95°C for 5 minutes, followed by 30 cycles of denaturation at 98°C for 20 seconds, annealing at 52, 54, and 56°C for 15 seconds, and extension at 72°C for 30 seconds. A final extension was performed at 72°C for 5 minutes. PCR products were visualized on agarose gels to confirm successful amplification, and samples showing weak or absent bands were re-amplified under the same conditions before downstream processing. Amplicons obtained from the three annealing temperature treatments (52, 54, and 56 °C) were combined for each sample to generate a single pooled product before indexing PCR.

Indexing PCR was performed using dual barcoded/indexing primers in a 30 µL reaction volume. Each reaction contained 15.00 µL Phusion Flash Master Mix (ThermoFisher F548), 0.563 µL each of forward and reverse indexing primers, 0.36 µL BSA (20 mg mL⁻¹; NEB), 10.52 µL DNase-free water, and 3.00 µL template DNA. Cycling conditions consisted of an initial denaturation at 95 °C for 10 s, followed by 9 cycles of denaturation at 98 °C for 15 s, annealing at 50 °C for 5 s, and extension at 72 °C for 20 s, with a final extension at 72 °C for 1 min and a hold at 4 °C. Indexed PCR products were verified by gel electrophoresis prior to purification. Dual-indexed Illumina TruSeq barcodes were used for multiplexing. Reverse barcode sequences correspond to the reverse-complement format required for Illumina demultiplexing.

*Sequence Processing and Bioinformatics Workflow*

Raw fungal ITS amplicon sequences were processed using the QIIME 2 platform (version 2024.2; Bolyen et al., 2019) on a high-performance computing environment (CREST cluster, Texas A&M University-Corpus Christi). All commands were executed using default parameters unless otherwise specified. Due to low R2 quality (typical for fungi), only R1 reads were used. Sequence data were imported into QIIME 2 using the qiime tools import command with the Casava 1.8 single-lane per sample format. Initial sequence quality, read length distribution, and sequencing depth were assessed using qiime demux summarize. Primers were trimmed using the cutadapt plugin implemented in QIIME 2 via qiime cutadapt trim-single. Forward and reverse ITS primers were removed using the --p-front parameter and --p-adapter to remove residual reverse-complement primer sequences when present. Reads lacking detectable primer sequences were discarded (--p-discard-untrimmed). Additional trimming parameters included a maximum error rate of 0.1 and a minimum overlap of 5 bp. Trimmed reads were subsequently evaluated using qiime demux summarize.

Denoising, quality filtering, chimera removal, and amplicon sequence variant (ASV) inference were performed using the DADA2 algorithm implemented in QIIME 2 via qiime dada2 denoise-single. Based on quality profiles, reads were trimmed by removing the first 10 bases (--p-trim-left 10) and truncated at 220 bases (--p-trunc-len 220). Additional quality-filtering parameters included truncation at a quality score of 2 (--p-trunc-q 2), a maximum expected error threshold of 2.0 (--p-max-ee 2.0), consensus-based chimera detection (--p-chimera-method consensus), and a minimum fold-parent over-abundance threshold of 1.0.

Taxonomic classification of ASVs was performed using a Naïve Bayes classifier trained against the UNITE fungal ITS reference database (99% similarity threshold; Abarenkov et al., 2023). Reference sequences and taxonomy files were imported into QIIME 2 as FeatureData[Sequence] and FeatureData[Taxonomy], respectively. A classifier was trained using qiime feature-classifier fit-classifier-naive-bayes. Representative ASV sequences were classified using qiime feature-classifier classify-sklearn with default parameters.

**References**

Abarenkov, K., Zirk, A., Piirmann, T., Pöhönen, R., Ivanov, F., Nilsson, R. H., & Kõljalg, U. (2023). *UNITE QIIME release for Fungi* (Version 18.07.2023) [Data set]. UNITE Community. <https://doi.org/10.15156/BIO/2938079>

Barbier, E. B., Hacker, S. D., Kennedy, C., Koch, E. W., Stier, A. C., & Silliman, B. R. (2011). The value of estuarine and coastal ecosystem services. *Ecological Monographs, 81*(2), 169–193. https://doi.org/10.1890/10-1510.1

Bolyen, E., Rideout, J. R., Dillon, M. R., Bokulich, N. A., Abnet, C. C., Al-Ghalith, G. A., Alexander, H., Alm, E. J., Arumugam, M., Asnicar, F., Bai, Y., Bisanz, J. E., Bittinger, K., Brejnrod, A., Brislawn, C. J., Brown, C. T., Callahan, B. J., Caraballo-Rodríguez, A. M., Chase, J., … Caporaso, J. G. (2019). Reproducible, interactive, scalable and extensible microbiome data science using QIIME 2. *Nature Biotechnology, 37*(8), 852–857. https://doi.org/10.1038/s41587-019-0209-9

Lumibao, C. Y., Formel, S., Elango, V., Pardue, J. H., Blum, M., & Van Bael, S. A. (2018). Persisting responses of salt marsh fungal communities to the Deepwater Horizon oil spill. *Science of the Total Environment, 642*, 904–913. https://doi.org/10.1016/j.scitotenv.2018.06.077

Vahsen, M. L., Blum, M. J., Megonigal, J. P., Emrich, S. J., Holmquist, J. R., Stiller, B., Todd-Brown, K. E. O., & McLachlan, J. S. (2023). Rapid plant trait evolution can alter coastal wetland resilience to sea level rise. *Science, 379*(6630), 393–398. https://doi.org/10.1126/science.abq0595

Vahsen, M. L., Kleiner, H. S., Kodak, H., Summers, J. L., Vahsen, W. L., Blum, M. J., Megonigal, J. P., & McLachlan, J. S. (2023). Complex eco-evolutionary responses of a foundational coastal marsh plant to global change. *New Phytologist, 240*(5), 2121–2136. https://doi.org/10.1111/nph.19117

Zedler, J. B., & Kercher, S. (2005). Wetland resources: Status, trends, ecosystem services, and restorability. *Annual Review of Environment and Resources, 30*, 39–74. https://doi.org/10.1146/annurev.energy.30.050504.144248

**ADDITIONAL RESULTS**

*Dispersion tests across all plants (H1)*

Pairwise PERMANOVA confirmed compositional differences between planted and unplanted soils (R² = 0.022, *P* < 0.001), between nitrogen treatments (R² = 0.009, *P* = 0.028), and between marsh microbe treatments (R² = 0.172, P < 0.001). Further tests of multivariate dispersion indicated significant heterogeneity for plant presence (F₁,₂₀₉ = 6.92, *P* = 0.010) and marsh microbe (F₁,₂₀₉ = 277.84, *P* < 0.001), but not for nitrogen treatments (F₁,₂₀₉ = 0.00, *P* = 0.991).

For guild level analyses, pairwise tests confirmed overall differences between planted and unplanted soils (*P* = 0.003), between nitrogen treatments (*P* = 0.0134), and between marsh microbe inoculation treatments (*P* < 0.001). Within-treatment comparisons further showed that the plant effect was significant only in the control treatment (*P* < 0.001) and not under marsh microbe inoculation (*P* = 0.246), whereas nitrogen effects were detected only under marsh microbe inoculation (*P* < 0.001) and not in control soils (*P* = 0.110). Tests of multivariate dispersion indicated no significant heterogeneity for plant presence (PERMDISP: F₁,₂₀₉ = 0.20, *P* = 0.662), but significant differences for nitrogen (PERMDISP: F₁,₂₀₉ = 4.97, *P* = 0.025) and marsh microbe (PERMDISP: F₁,₂₀₉ = 11.81, *P* < 0.001).

*Multimodel analyses for P+M+ dataset only (H2)*

Multi-model inference for P+M+ only indicated that nitrogen and plant trait variation jointly influenced fungal ASV *ENSpie* diversity, although overall explanatory power was modest. Model-averaged results identified R:S (Σw = 0.57) as the most consistently retained predictor among traits, whereas all others showed substantially lower importance (Σw ≤ 0.30). Conditional model-averaged estimates indicated a marginal negative effect of R:S on *ENSpie* (β = -0.090 ± 0.049, *P* = 0.066) and a weak negative effect of nitrogen (β = -0.185 ± 0.105, *P* = 0.077), while SDi, SLA, SN, BG, IW, and age cohort showed no detectable influence (all *P* ≥ 0.29). Across the set of competitive models (ΔAIC ≤ 4), explanatory power remained low (conditional R² range = 0.063–0.137; mean = 0.10). The best-supported single model retained nitrogen and R:S as predictors of *ENSpie* diversity. Nitrogen exerted a significant negative effect (β = -0.229 ± 0.094, *P* = 0.015), whereas R:S not significant (β = -0.086 ± 0.047, *P* = 0.070). The random effect of genotype explained negligible variance (σ² ≈ 0), indicating limited genotype-level contribution to diversity patterns in this model.

Pearson correlation analysis showed that SH was highly correlated with other plant traits (r = 0.34–0.63), thus it was excluded to improve model stability.

At the guild level, plant age cohort was the primary biological predictor of fungal *ENSpie* diversity, whereas nitrogen and plant trait variation contributed little explanatory power. Plant age cohort showed relatively high model importance (Σw = 0.84) and exerted a significant negative effect on *ENSpie* (β = -0.178 ± 0.078, *P* = 0.022), indicating lower fungal diversity associated with the modern cohort. IW and all measured plant traits (BG, RS, SN, SLA, SDi) had low importance (Σw ≤ 0.20) and showed no detectable influence on fungal diversity (all *P* ≥ 0.39). Across competitive models (ΔAIC ≤ 4), explanatory power remained low (conditional R² range = 0.006–0.105; mean = 0.073). The best-supported single model retained plant age cohort and nitrogen as predictors of *ENSpie* diversity. Plant age cohort exerted a significant negative effect (β = -0.171 ± 0.074, *P* = 0.022), confirming reduced fungal diversity in soils associated with the modern cohort, whereas nitrogen had no detectable influence (β = 0.046 ± 0.072, *P* = 0.523).

**Figure S1.** Mean relative abundances of fungal phyla (a, b) and classes (c, d) across PSM treatments (P+M+, P-M+, P+M-, P-M-) under nitrogen inputs (N-: Low; N+: High) and plant age cohorts (Ancestral, Modern). Ancestral and modern plant cohorts are shown separately from unplanted soils. Relative abundance was calculated for each sample and averaged across samples within each treatment group. Only the top taxa are shown, with remaining taxa grouped as “Other.”

**Figure S2.** Mean relative abundances of fungal ecological guilds under nitrogen treatments (Low and High) across different PSM treatments (P+M+, P-M+, P+M-, P-M-) for ancestral and modern plant cohorts (a), and unplanted soils (b). Relative abundance was calculated for each sample and averaged across samples within each treatment group. Only the top 10 most abundant guilds are shown, with remaining guilds grouped as “Other.”

**Figure S3.** Estimated marginal means (± SE) of *ENSpie* showing the stepwise plant effect across nitrogen treatments (Low and High). Plant effects were calculated using the stepwise difference approach described in the Methods, where fungal diversity of plants with marsh microbes (P+M+) was first contrasted with plant controls lacking marsh microbes (P+M-) (Diff 1), and fungal diversity of soils with microbes but no plants (P-M+) was contrasted with microbe controls (P-M-) (Diff 2) within each nitrogen treatment and replicate. The final plant effect was obtained by subtracting Diff 2 from Diff 1, thereby accounting for background microbial communities independent of plants. Circles represent ASV-based estimates and triangles represent guild-based estimates. The horizontal dashed line indicates zero, corresponding to no net plant effect after background subtraction.

**Table S1.** Pairwise comparisons of estimated marginal means (EMMs) for *ENSpie* across plant presence and nitrogen input (H1). Values are Tukey-adjusted pairwise comparisons of back-transformed estimated marginal means averaged across marsh microbe levels. Bold P-values indicate *P* < 0.05; df = degrees of freedom.

| **Comparison** | **Ratio** | **SE** | **df** | **t value** | **P value** |
| --- | --- | --- | --- | --- | --- |
| **Within Plant presence (Nitrogen Input effect)** |  |  |  |  |  |
| P-: Low / High | 0.863 | 0.101 | 203 | -1.262 | 0.208 |
| P+: Low / High | 1.222 | 0.119 | 203 | 2.049 | **0.042** |
| **Within Nitrogen Input (Plant presence effect)** |  |  |  |  |  |
| Low: P- / P+ | 0.607 | 0.067 | 203 | -4.496 | **<0.001** |
| High: P- / P+ | 0.86 | 0.09 | 203 | -1.441 | 0.151 |

**Table S2.** PERMANOVA results testing the effects of plant traits, plant age cohort, and marsh microbe and nitrogen treatment on fungal community composition at the ASV (top) and guild (bottom) levels. Significant effects in bold (* P < 0.05); df = degrees of freedom.

| ***ASV*** |  |  |  |  |
| --- | --- | --- | --- | --- |
| **Term** | **Df** | **R²** | **F** | **P-value** |
| Plant Age Cohort | 1 | 0.014 | 1.832 | **0.047** |
| Marsh Microbe × Nitrogen Input | 1 | 0.014 | 1.869 | **0.039** |
| BG | 1 | 0.012 | 1.541 | 0.106 |
| SN | 1 | 0.011 | 1.402 | 0.133 |
| IW | 1 | 0.009 | 1.153 | 0.249 |
| SDi | 1 | 0.006 | 0.857 | 0.559 |
| R:S | 1 | 0.005 | 0.713 | 0.686 |
| SLA | 1 | 0.004 | 0.517 | 0.947 |
| Residual | 94 | 0.708 |  |  |
| Total | 104 | 1 |  |  |
| ***Guild*** |  |  |  |  |
| **Term** | **Df** | **R²** | **F** | **P-value** |
| Plant Age Cohort | 1 | 0.022 | 3.057 | **0.019** |
| SDi | 1 | 0.013 | 1.753 | 0.141 |
| IW | 1 | 0.011 | 1.515 | 0.191 |
| Marsh Microbe × Nitrogen Input | 1 | 0.010 | 1.334 | 0.251 |
| BG | 1 | 0.008 | 1.081 | 0.317 |
| R:S | 1 | 0.005 | 0.695 | 0.485 |
| SLA | 1 | 0.004 | 0.492 | 0.710 |
| SN | 1 | 0.003 | 0.419 | 0.778 |
| Residual | 94 | 0.673 |  |  |
| Total | 104 | 1 |  |  |

**Table S3.** Indicator ASVs identified by multilevel pattern analysis (IndVal.g). Indicator species analysis was performed using multipatt (package indicspecies) with the IndVal.g association function. ASVs were tested for significant association with the nitrogen Input × plant age cohort combined factor. Significance was assessed using 9999 permutations (α = 0.05).

| **Treatment** | **ASV ID** | **Taxonomy (p, c, o)** | **IndVal** | **A** | **B** | **P-value** |
| --- | --- | --- | --- | --- | --- | --- |
| Low × Ancestral | ASV_2136 | p__Ascomycota; c__Pezizomycetes; o__Pezizales | 0.657 | 0.6266 | 0.6897 | 0.0023 |
| Low × Ancestral | ASV_595 | p__Ascomycota; c__Sordariomycetes; o__Sordariales | 0.643 | 0.5213 | 0.7931 | 0.0031 |
| Low × Ancestral | ASV_2902 | p__Ascomycota; c__Dothideomycetes; o__Venturiales | 0.614 | 0.5469 | 0.6897 | 0.0148 |
| Low × Ancestral | ASV_605 | p__Ascomycota; c__Sordariomycetes; o__Sordariales | 0.548 | 0.4592 | 0.6552 | 0.0433 |
| Low × Ancestral | ASV_2257 | p__Ascomycota; c__Saccharomycetes; o__Saccharomycetales | 0.528 | 0.735 | 0.3793 | 0.037 |
| Low × Ancestral | ASV_935 | p__Ascomycota; c__Sordariomycetes; o__Hypocreales | 0.509 | 0.6839 | 0.3793 | 0.012 |
| Low × Ancestral | ASV_3110 | p__Basidiomycota; c__Agaricomycetes; o__Hymenochaetales | 0.5 | 0.905 | 0.2759 | 0.0046 |
| Low × Ancestral | ASV_3106 | p__Basidiomycota; c__Agaricomycetes; o__Hymenochaetales | 0.488 | 0.9854 | 0.2414 | 0.0019 |
| Low × Ancestral | ASV_2640 | p__Fungi_phy_Incertae_sedis; c__Fungi_cls_Incertae_sedis; o__Fungi_ord_Incertae_sedis | 0.467 | 0.9034 | 0.2414 | 0.003 |
| Low × Ancestral | ASV_2122 | p__Ascomycota; c__Eurotiomycetes; o__Chaetothyriales | 0.455 | 1 | 0.2069 | 0.001 |
| Low × Ancestral | ASV_3109 | p__Basidiomycota; c__Agaricomycetes; o__Hymenochaetales | 0.451 | 0.9822 | 0.2069 | 0.0167 |
| Low × Ancestral | ASV_3111 | p__Basidiomycota; c__Agaricomycetes; o__Hymenochaetales | 0.449 | 0.9738 | 0.2069 | 0.0085 |
| Low × Ancestral | ASV_2869 | p__Ascomycota; c__Dothideomycetes; o__Venturiales | 0.448 | 0.5827 | 0.3448 | 0.0303 |
| Low × Ancestral | ASV_2870 | p__Ascomycota; c__Dothideomycetes; o__Venturiales | 0.445 | 0.6373 | 0.3103 | 0.0327 |
| Low × Ancestral | ASV_2907 | p__Ascomycota; c__Dothideomycetes; o__Venturiales | 0.412 | 0.8187 | 0.2069 | 0.0143 |
| Low × Ancestral | ASV_1091 | p__Ascomycota; c__Sordariomycetes; o__Xylariales | 0.408 | 0.9668 | 0.1724 | 0.0173 |
| Low × Ancestral | ASV_2062 | p__Ascomycota; c__Saccharomycetes; o__Saccharomycetales | 0.396 | 0.9098 | 0.1724 | 0.0146 |
| Low × Ancestral | ASV_1041 | p__Ascomycota; c__Sordariomycetes; o__Sordariomycetes_ord_Incertae_sedis | 0.322 | 1 | 0.1034 | 0.0285 |
| Low × Ancestral | ASV_3525 | p__Ascomycota; c__Sordariomycetes; o__Sordariales | 0.322 | 1 | 0.1034 | 0.0303 |
| Low × Ancestral | ASV_3548 | p__Basidiomycota; c__Agaricomycetes; o__Sebacinales | 0.322 | 1 | 0.1034 | 0.0309 |
| High × Ancestral | ASV_2936 | p__Ascomycota; c__Eurotiomycetes; o__Eurotiales | 0.407 | 0.7175 | 0.2308 | 0.0361 |
| Low × Modern | ASV_1344 | p__Ascomycota; c__Sordariomycetes; o__Sordariales | 0.462 | 0.9621 | 0.2222 | 0.0129 |
| Low × Modern | ASV_1205 | p__Ascomycota; c__Sordariomycetes; o__Sordariomycetes_ord_Incertae_sedis | 0.46 | 0.634 | 0.3333 | 0.0217 |
| Low × Modern | ASV_1349 | p__Ascomycota; c__Sordariomycetes; o__Sordariales | 0.436 | 0.8545 | 0.2222 | 0.0068 |
| Low × Modern | ASV_1215 | p__Ascomycota; c__Sordariomycetes; o__Sordariomycetes_ord_Incertae_sedis | 0.408 | 1 | 0.1667 | 0.0045 |
| Low × Modern | ASV_2511 | p__Ascomycota; c__Dothideomycetes; o__Pleosporales | 0.408 | 1 | 0.1667 | 0.0047 |
| Low × Modern | ASV_2329 | p__Fungi_phy_Incertae_sedis; c__Fungi_cls_Incertae_sedis; o__Fungi_ord_Incertae_sedis | 0.405 | 0.9848 | 0.1667 | 0.0045 |
| Low × Modern | ASV_2309 | p__Fungi_phy_Incertae_sedis; c__Fungi_cls_Incertae_sedis; o__Fungi_ord_Incertae_sedis | 0.404 | 0.9814 | 0.1667 | 0.0188 |
| Low × Modern | ASV_385 | p__Ascomycota; c__Dothideomycetes; o__Pleosporales | 0.398 | 0.95 | 0.1667 | 0.0099 |
| Low × Modern | ASV_332 | k__Fungi | 0.389 | 0.9056 | 0.1667 | 0.0322 |
| Low × Modern | ASV_3319 | k__Fungi | 0.382 | 0.6573 | 0.2222 | 0.0315 |
| Low × Modern | ASV_2348 | p__Rozellomycota; c__Rozellomycota_cls_Incertae_sedis; o__Rozellomycota_ord_Incertae_sedis | 0.38 | 0.8657 | 0.1667 | 0.0135 |
| Low × Modern | ASV_2028 | p__Ascomycota; c__Pezizomycetes; o__Pezizales | 0.37 | 0.8194 | 0.1667 | 0.0233 |
| Low × Modern | ASV_1973 | k__Fungi | 0.363 | 0.7899 | 0.1667 | 0.0227 |
| Low × Modern | ASV_63 | p__Chytridiomycota; c__Cladochytriomycetes; o__Cladochytriales | 0.333 | 1 | 0.1111 | 0.0242 |
| Low × Modern | ASV_331 | k__Fungi | 0.333 | 1 | 0.1111 | 0.0292 |
| Low × Modern | ASV_1408 | p__Ascomycota; c__Sordariomycetes; o__Hypocreales | 0.333 | 1 | 0.1111 | 0.0309 |
| Low × Modern | ASV_1409 | p__Ascomycota; c__Sordariomycetes; o__Hypocreales | 0.333 | 1 | 0.1111 | 0.0292 |
| Low × Modern | ASV_1524 | k__Fungi | 0.333 | 1 | 0.1111 | 0.0288 |
| Low × Modern | ASV_1531 | k__Fungi | 0.333 | 1 | 0.1111 | 0.0302 |
| Low × Modern | ASV_1558 | k__Fungi | 0.333 | 1 | 0.1111 | 0.0291 |
| Low × Modern | ASV_1618 | k__Fungi | 0.333 | 1 | 0.1111 | 0.0257 |
| Low × Modern | ASV_1647 | k__Fungi | 0.333 | 1 | 0.1111 | 0.0285 |
| Low × Modern | ASV_2035 | p__Ascomycota; c__Pezizomycetes; o__Pezizales | 0.333 | 1 | 0.1111 | 0.0283 |
| Low × Modern | ASV_2322 | p__Fungi_phy_Incertae_sedis; c__Fungi_cls_Incertae_sedis; o__Fungi_ord_Incertae_sedis | 0.333 | 1 | 0.1111 | 0.0268 |
| Low × Modern | ASV_3453 | p__Basidiomycota; c__Malasseziomycetes; o__Malasseziales | 0.333 | 1 | 0.1111 | 0.0298 |
| Low × Modern | ASV_3597 | k__Fungi | 0.333 | 1 | 0.1111 | 0.0283 |
| Low × Modern | ASV_112 | k__Fungi | 0.332 | 0.9931 | 0.1111 | 0.0281 |
| Low × Modern | ASV_393 | p__Ascomycota; c__Dothideomycetes; o__Pleosporales | 0.327 | 0.9628 | 0.1111 | 0.0412 |
| Low × Modern | ASV_3463 | p__Basidiomycota; c__Malasseziomycetes; o__Malasseziales | 0.319 | 0.9186 | 0.1111 | 0.0484 |
| High × Modern | ASV_2667 | p__Ascomycota; c__Eurotiomycetes; o__Eurotiales | 0.383 | 0.6951 | 0.2105 | 0.0252 |
| High × Modern | ASV_2298 | p__Fungi_phy_Incertae_sedis; c__Fungi_cls_Incertae_sedis; o__Fungi_ord_Incertae_sedis | 0.377 | 0.9023 | 0.1579 | 0.0343 |
| High × Modern | ASV_793 | p__Ascomycota; c__Sordariomycetes; o__Microascales | 0.369 | 0.8646 | 0.1579 | 0.0241 |
| High × Modern | ASV_3304 | p__Basidiomycota; c__Agaricomycetes; o__Agaricomycetes_ord_Incertae_sedis | 0.35 | 0.7738 | 0.1579 | 0.0343 |

**Table S4.** Indicator ecological guilds identified by multilevel pattern analysis (IndVal.g). Indicator guild analysis was conducted using multipatt (indicspecies) with the IndVal.g association function. Guilds were tested for significant association with (a) nitrogen input (Low vs High), (b) plant age cohort (Ancestral vs Modern), (c) plant genotype, and (d) the nitrogen input × plant age cohort combined factor. Significance was assessed using 9999 permutations (α = 0.05).

| **Treatment** | **Guild** | **IndVal** | **A** | **B** | **P-value** |
| --- | --- | --- | --- | --- | --- |
| ***a. Nitrogen input indicator Guilds (Low vs High)*** | |  |  |  |  |
| Low N | Animal Parasite-Undefined Saprotroph | 0.723 | 0.767 | 0.681 | 0.011 |
| Low N | Animal Pathogen-Plant Pathogen-Undefined Saprotroph | 0.618 | 0.855 | 0.447 | 0.038 |
| Low N | Wood Saprotroph | 0.595 | 0.926 | 0.383 | 0.010 |
| Low N | Plant Pathogen-Undefined Saprotroph | 0.375 | 0.947 | 0.149 | 0.010 |
| Low N | Plant Saprotroph-Wood Saprotroph | 0.372 | 0.928 | 0.149 | 0.009 |
| Low N | Animal Pathogen-Endophyte-Fungal Parasite-Undefined Saprotroph | 0.357 | 1.000 | 0.128 | 0.007 |
| High N | Dung Saprotroph | 0.651 | 0.848 | 0.500 | 0.012 |
| High N | Animal Pathogen-Endophyte-Plant Pathogen-Plant Saprotroph-Undefined Saprotroph-Wood Saprotroph | 0.486 | 0.913 | 0.259 | 0.031 |
| ***b. Plant age cohort indicator Guilds (Ancestral vs Modern)*** | |  |  |  |  |
| Modern | Animal Pathogen-Undefined Saprotroph | 0.584 | 0.840 | 0.405 | 0.001 |
| Modern | Dung Saprotroph-Endophyte-Undefined Saprotroph | 0.285 | 1.000 | 0.081 | 0.045 |
| Modern | Ectomycorrhizal | 0.285 | 1.000 | 0.081 | 0.040 |
| ***c. Plant genotype indicator Guilds*** | |  |  |  |  |
| M1 | Plant Pathogen | 0.480 | 0.690 | 0.333 | 0.026 |
| M1 | Ectomycorrhizal | 0.449 | 0.909 | 0.222 | 0.042 |
| ***d. Nitrogen input × Plant age cohort indicator Guilds*** | |  |  |  |  |
| Low x Ancestral | Animal Parasite-Undefined Saprotroph | 0.655 | 0.592 | 0.724 | 0.011 |
| Low x Ancestral | Wood Saprotroph | 0.631 | 0.889 | 0.448 | 0.004 |
| Low x Ancestral | Animal Pathogen-Endophyte-Fungal Parasite-Undefined Saprotroph | 0.455 | 1.000 | 0.207 | 0.002 |
| Low x Modern | Ectomycorrhizal | 0.319 | 0.914 | 0.111 | 0.048 |
| High x Modern | Bryophyte Parasite-Plant Saprotroph-Wood Saprotroph | 0.326 | 0.672 | 0.158 | 0.034 |

**Table S5.** Structural equation model (SEM) results for fungal diversity (*ENSpie*). Standardized SEM coefficients are shown for ASV-based (top panel) and ecological guild-based (bottom) datasets. Predictors include plant traits and experimental factors: marsh microbe, nitrogen input, and plant age cohort. Values reported are unstandardized estimates, standard errors (SE), test statistics (z), P values, and standardized coefficients (Std. Estimate). Bold P-values indicate *P* < 0.05.

| ***ASV*** |  |  |  |  |  |  |
| --- | --- | --- | --- | --- | --- | --- |
| **Response** | **Predictor** | **Estimate** | **Std. Error** | **Test Statistic** | **P value** | **Std. Estimate** |
| RS | IW | 0.052 | 0.119 | 0.435 | 0.665 | 0.040 |
| RS | Marsh Microbe | -0.172 | 0.214 | -0.804 | 0.423 | -0.074 |
| RS | Nitrogen Input | -0.758 | 0.210 | -3.618 | **<0.001** | -0.335 |
| RS | Age Cohort | -0.526 | 0.219 | -2.408 | **0.018** | -0.223 |
| BG | IW | 0.473 | 0.177 | 2.667 | **0.009** | 0.247 |
| BG | Marsh Microbe | -0.587 | 0.321 | -1.830 | 0.070 | -0.170 |
| BG | Nitrogen Input | -0.430 | 0.314 | -1.371 | 0.173 | -0.127 |
| BG | Age Cohort | -0.678 | 0.327 | -2.074 | **0.041** | -0.193 |
| SDi | IW | 0.041 | 0.043 | 0.963 | 0.338 | 0.096 |
| SDi | Marsh Microbe | -0.019 | 0.077 | -0.253 | 0.801 | -0.025 |
| SDi | Nitrogen Input | 0.034 | 0.075 | 0.450 | 0.654 | 0.045 |
| SDi | Age Cohort | 0.034 | 0.078 | 0.438 | 0.663 | 0.044 |
| SLA | IW | -0.086 | 0.091 | -0.942 | 0.349 | -0.092 |
| SLA | Marsh Microbe | -0.097 | 0.165 | -0.590 | 0.556 | -0.058 |
| SLA | Nitrogen Input | 0.267 | 0.161 | 1.657 | 0.101 | 0.162 |
| SLA | Age Cohort | 0.279 | 0.168 | 1.664 | 0.099 | 0.162 |
| SN | IW | 0.185 | 0.086 | 2.157 | **0.033** | 0.208 |
| SN | Marsh Microbe | -0.303 | 0.155 | -1.954 | 0.054 | -0.188 |
| SN | Nitrogen Input | 0.082 | 0.152 | 0.539 | 0.591 | 0.052 |
| SN | Age Cohort | 0.062 | 0.158 | 0.393 | 0.695 | 0.038 |
| *ENSpie* | IW | 0.110 | 0.062 | 1.773 | 0.080 | — |
| *ENSpie* | RS | 0.037 | 0.091 | 0.405 | 0.687 | — |
| *ENSpie* | BG | -0.034 | 0.091 | -0.373 | 0.710 | — |
| *ENSpie* | SDi | -0.109 | 0.172 | -0.634 | 0.528 | — |
| *ENSpie* | SLA | 0.067 | 0.069 | 0.970 | 0.334 | — |
| *ENSpie* | SN | -0.030 | 0.178 | -0.168 | 0.867 | — |
| *ENSpie* | Marsh Microbe | 0.673 | 0.111 | 6.062 | **<0.001** | — |
| *ENSpie* | Nitrogen Input | -0.236 | 0.114 | -2.077 | **0.041** | — |
| *ENSpie* | Age Cohort | -0.105 | 0.118 | -0.890 | 0.376 | — |
| ~~BG | ~~RS | 0.396 | — | 4.351 | **<0.001** | 0.396 |
| ~~SDi | ~~BG | 0.386 | — | 4.231 | **<0.001** | 0.386 |
| ~~SN | ~~BG | 0.756 | — | 11.652 | **<0.001** | 0.756 |
| ~~SLA | ~~SDi | 0.293 | — | 3.097 | **0.001** | 0.293 |
| ~~SN | ~~SLA | 0.186 | — | 1.915 | **0.029** | 0.186 |
| ~~SN | ~~SDi | 0.527 | — | 6.260 | **<0.001** | 0.527 |
| ~~SDi | ~~RS | -0.169 | — | -1.736 | **0.043** | -0.169 |
| ***Guild*** |  |  |  |  |  |  |
| **Response** | **Predictor** | **Estimate** | **Std.Error** | **Crit.Value** | **P.Value** | **Std.Estimate** |
| RS | IW | 0.052 | 0.119 | 0.435 | 0.665 | 0.040 |
| RS | Marsh Microbe | -0.172 | 0.214 | -0.804 | 0.423 | -0.074 |
| RS | Nitrogen Input | -0.758 | 0.210 | -3.618 | **<0.001** | -0.335 |
| RS | Age Cohort | -0.526 | 0.219 | -2.408 | **0.018** | -0.223 |
| BG | IW | 0.473 | 0.177 | 2.667 | **0.009** | 0.247 |
| BG | Marsh Microbe | -0.587 | 0.321 | -1.830 | 0.070 | -0.170 |
| BG | Nitrogen Input | -0.430 | 0.314 | -1.371 | 0.173 | -0.127 |
| BG | Age Cohort | -0.678 | 0.327 | -2.074 | **0.041** | -0.193 |
| SDi | IW | 0.041 | 0.043 | 0.963 | 0.338 | 0.096 |
| SDi | Marsh Microbe | -0.019 | 0.077 | -0.253 | 0.801 | -0.025 |
| SDi | Nitrogen Input | 0.034 | 0.075 | 0.450 | 0.654 | 0.045 |
| SDi | Age Cohort | 0.034 | 0.078 | 0.438 | 0.663 | 0.044 |
| SLA | IW | -0.086 | 0.091 | -0.942 | 0.349 | -0.092 |
| SLA | Marsh Microbe | -0.097 | 0.165 | -0.590 | 0.556 | -0.058 |
| SLA | Nitrogen Input | 0.267 | 0.161 | 1.657 | 0.101 | 0.162 |
| SLA | Age Cohort | 0.279 | 0.168 | 1.664 | 0.099 | 0.162 |
| SN | IW | 0.185 | 0.086 | 2.157 | **0.033** | 0.208 |
| SN | Marsh Microbe | -0.303 | 0.155 | -1.954 | 0.054 | -0.188 |
| SN | Nitrogen Input | 0.082 | 0.152 | 0.539 | 0.591 | 0.052 |
| SN | Age Cohort | 0.062 | 0.158 | 0.393 | 0.695 | 0.038 |
| *ENSpie* | IW | 0.053 | 0.033 | 1.584 | 0.117 | - |
| *ENSpie* | RS | 0.031 | 0.049 | 0.636 | 0.526 | - |
| *ENSpie* | BG | -0.057 | 0.049 | -1.165 | 0.247 | - |
| *ENSpie* | SDi | -0.090 | 0.092 | -0.979 | 0.330 | - |
| *ENSpie* | SLA | 0.024 | 0.037 | 0.660 | 0.511 | - |
| *ENSpie* | SN | 0.082 | 0.095 | 0.860 | 0.392 | - |
| *ENSpie* | Marsh Microbe | 0.369 | 0.059 | 6.233 | **<0.001** | - |
| *ENSpie* | Nitrogen Input | 0.021 | 0.061 | 0.346 | 0.730 | - |
| *ENSpie* | Age Cohort | -0.189 | 0.063 | -2.990 | **0.004** | - |
| ~~BG | ~~RS | 0.396 | - | 4.351 | **<0.001** | 0.396 |
| ~~SDi | ~~BG | 0.386 | - | 4.231 | **<0.001** | 0.386 |
| ~~SN | ~~BG | 0.756 | - | 11.652 | **<0.001** | 0.756 |
| ~~SLA | ~~SDi | 0.293 | - | 3.097 | **0.001** | 0.293 |
| ~~SN | ~~SLA | 0.186 | - | 1.915 | **0.029** | 0.186 |
| ~~SN | ~~SDi | 0.527 | - | 6.260 | **<0.001** | 0.527 |
| ~~SDi | ~~RS | -0.169 | - | -1.736 | **0.043** | -0.169 |
