# Supplementary material for: Rhizosphere Fungal Community Responses to Nitrogen Addition Reflect Genotypic Versus Trait Variation in the Marsh Sedge Schoenoplectus americanus Over Time
Source: Mol Ecol. 2026 Oct 14;35(20):e70588. doi: 10.1111/mec.70588 (PMC13667335; doi:10.1111/mec.70588)
Supplement: Supplementary file 2 [file MEC-35-e70588-s002.docx]

**Supporting Information 2 for:**

**Rhizosphere fungal community responses to elevated nitrogen reflect genotypic versus trait variation in the marsh sedge *Schoenoplectus americanus* over time**

Yue Liu^1^, Michael J. Blum^2^ and Candice Y. Lumibao^1^

^1^Department of Life Sciences, Texas A&M University – Corpus Christi, Corpus Christi, Texas, USA

^2^Department of Ecology & Evolution, University of Tennessee – Knoxville, Knoxville, Tennessee, USA

**Table of Contents:**

| **Results** | Page 2 |
| --- | --- |
| **Figures S4** | Page 4 |
| **Figures S5** | Page 5 |
| **Tables S6** | Page 6 |
| **Tables S7** | Page 7 |
| **Tables S8** | Page 8 |
| **Tables S9** | Page 9 |
|  |  |

**SHANNON RESULTS**

*Overall influence of plant presence on soil fungal Shannon diversity (H1)*

Generalized linear models showed significant effects of plant presence (F₁,₂₀₃ = 14.08, *P* < 0.001; Table S6, Figure S4) and marsh microbe inoculation (F₁,₂₀₃ = 262.55, P < 0.001; Table S6, Figure S4) on fungal Shannon diversity, with a significant plant × microbe interaction (F₁,₂₀₃ = 6.00, P = 0.015; Table S6). N input had no effect on Shannon diversity (F₁,₂₀₃ = 0.21, P = 0.650; Table S6), and interactions involving N were not significant (Table S6). Post hoc comparisons indicated that marsh microbe inoculation increased Shannon diversity in both bulk and rhizosphere soils (P-: estimate = -1.34 ± 0.11, t = -12.27, P < 0.001; P+: estimate = -1.00 ± 0.09, t = -10.95, P < 0.001; Table S7). The effect of plant presence was detected only under the control treatment (P+M- vs. P-M-), where rhizosphere soils exhibited higher Shannon diversity than bulk soils (estimate = -0.469 ± 0.114, t = -4.13, P < 0.001; Table S7), but no plant effect was observed under marsh microbe inoculation (estimate = -0.130 ± 0.086, t = -1.51, *P* = 0.132; Table S7).

Patterns for guild-level Shannon diversity differed from ASV-level responses. Plant presence was not significant (F₁,₂₀₁ = 0.69, P = 0.407; Table S6), and N input was not significant (F₁,₂₀₁ = 1.32, P = 0.252; Table S6) as well. However, marsh microbe inoculation had a strong effect on Shannon diversity (F₁,₂₀₁ = 120.86, P < 0.001; Table S6). Post hoc tests confirmed higher Shannon diversity under marsh microbe inoculation than in the control treatment in both bulk and rhizosphere soils (P-: estimate = -0.457 ± 0.074, t = -6.14, P < 0.001; P+: estimate = -0.561 ± 0.062, t = -9.07, *P* < 0.001; Table S7). No plant effect was detected within either microbe treatment level (M-: P = 0.671; M+: *P* = 0.215; Table S7).

*Influence of intraspecific S. americanus trait variation on rhizosphere fungal Shannon diversity across age cohorts (H2)*

Patterns based on ASV Shannon diversity provided partial support for H2, indicating that the influence of heritable plant trait variation on fungal diversity remained limited and context dependent. Multimodel inference identified marsh microbe inoculation and N input as the most consistently retained predictors (Σw = 1.00 for both; Table S8). Fungal Shannon diversity was significantly higher in inoculated soils compared to control soils regardless of N level (β = 0.82 ± 0.11, P < 0.001; Table S8). In contrast, N input had no significant effect on fungal Shannon diversity (β = -0.15 ± 0.11, P = 0.18; Table S8). Among plant traits, only initial plant weight (IW) showed a significant positive effect on fungal Shannon diversity, although with relatively low model importance (Σw = 0.22; β = 0.10 ± 0.05, P = 0.034; Table S8). Other predictors, including plant age cohort and the marsh microbe × N interaction, showed low importance and were not significant (Σw ≤ 0.10, P ≥ 0.24; Table S8). At the guild level, Shannon diversity was strongly increased by marsh microbe inoculation, while N and plant age cohort had no significant effect (Table S8).

Structural equation modeling indicated limited plant trait–mediated pathways linking N treatment to rhizosphere fungal Shannon diversity. The model showed good overall fit (χ² = 5.23, df = 3, p = 0.156; Fisher’s C = 8.15, df = 6, p = 0.227) and explained a moderate proportion of variation in Shannon diversity (R² = 0.45). Marsh microbe inoculation exerted a strong positive direct effect on Shannon diversity (β = 0.623, p < 0.001; Figure S5, Table S9), whereas N treatment showed no significant direct influence (p = 0.165; Figure S5, Table S9). IW had a weak but significant positive effect on Shannon diversity (β = 0.172, p = 0.034; Figure S5, Table S9). None of the measured plant traits significantly influenced Shannon diversity (all p ≥ 0.52; Figure S5, Table S9). N significantly reduced R:S (β = -0.335, p = 0.0005; Figure S5, Table S9), and plant age cohort influenced both R:S (β = -0.223, p = 0.018) and BG (β = -0.193, p = 0.041). IW was positively associated with BG (β = 0.247, p = 0.009) and SN (β = 0.208, p = 0.033) (Figure S5, Table S9). However, trait variation did not translate into indirect effects on Shannon diversity.

The guild-level SEM recovered similar patterns, with acceptable overall model fit (χ² = 5.23, df = 3, P = 0.156; Fisher’s C = 8.15, df = 6, P = 0.227) and explained a moderate proportion of variation in guild-level Shannon diversity (R² = 0.44). Marsh microbe inoculation exerted a strong positive direct effect on Shannon diversity (β = 0.636, p < 0.001; Table S9), whereas N treatment and age cohort showed no significant direct influence (p = 0.692; p = 0.054; Table S9). None of the measured plant traits showed significant direct associations with Shannon diversity (all p ≥ 0.13; Table S9).

**Figure S4.** Raw ASV-level Shannon (mean ± SE) across plant-soil-microbe (PSM) treatments under low and high N. Treatments were combinations of plant presence (P+/P-) and marsh microbe inoculation (M+/M-).

**Figure S5.** Piecewise structural equation model (SEM) describing factors influencing ASV-level rhizosphere Shannon diversity. The model quantifies direct and indirect effects of marsh microbe inoculation, N input, plant age cohort, and plant heritable traits on fungal diversity. Arrows represent significant causal paths (*P* < 0.05), and numbers indicate standardized path coefficients. Only significant relationships are depicted.

**Table S6.** Overall effects of plant presence, marsh microbe inoculation, and N Input on fungal ASV (top panel) and guild (bottom) diversity (Shannon) (H1). Type II analysis of deviance from Gamma GLMs (log link). Bold P-values indicate *P* < 0.05; df = degrees of freedom.

| ***ASV*** |  |  |  |  |
| --- | --- | --- | --- | --- |
| **Effect** | **df** | **Sum of Squares** | **F value** | **P value** |
| Plant presence | 1 | 3.352 | 14.08 | **<0.001** |
| Marsh microbe | 1 | 62.498 | 262.555 | **<0.001** |
| N Input | 1 | 0.049 | 0.206 | 0.65 |
| Plant presence × Marsh microbe | 1 | 1.427 | 5.996 | **0.015** |
| Plant presence × N Input | 1 | 0.079 | 0.33 | 0.566 |
| Marsh microbe × N Input | 1 | 0.168 | 0.707 | 0.401 |
| Plant presence × Marsh microbe × N Input | 1 | 0.265 | 1.115 | 0.292 |
| Residuals | 203 | 48.322 | — | — |
| ***Guild*** |  |  |  |  |
| **Effect** | **df** | **Sum of Squares** | **F value** | **P value** |
| Plant presence | 1 | 0.074 | 0.692 | 0.407 |
| Marsh microbe | 1 | 12.955 | 120.855 | **<0.001** |
| N Input | 1 | 0.141 | 1.319 | 0.252 |
| Plant presence × Marsh microbe | 1 | 0.101 | 0.945 | 0.332 |
| Plant presence × N Input | 1 | 0 | 0 | 0.989 |
| Marsh microbe × N Input | 1 | 0.079 | 0.737 | 0.392 |
| Plant presence × Marsh microbe × N Input | 1 | 0.215 | 2.007 | 0.158 |
| Residuals | 201 | 21.546 | — | — |

**Table S7.** Pairwise comparisons of estimated marginal means (EMMs) for Shannon across plant presence and marsh microbe inoculation conditions. Results are based on Gaussian GLMs (identity link). Values are Tukey-adjusted pairwise comparisons averaged across N input levels. Bold P-values indicate *P* < 0.05; df = degrees of freedom.

| **Comparison** | **Estimate** | **SE** | **df** | **t value** | **P value** |
| --- | --- | --- | --- | --- | --- |
| ***Within Plant presence (Marsh microbe effect)*** |  |  |  |  |  |
| P-: M^-^ - M+ | -1.34 | 0.109 | 203 | -12.267 | **<0.001** |
| P+: M^-^ - M+ | -1 | 0.091 | 203 | -10.952 | **<0.001** |
| ***Within Marsh microbe (Plant presence effect)*** |  |  |  |  |  |
| M-: P^-^ - P+ | -0.469 | 0.114 | 203 | -4.131 | **<0.001** |
| M+: P^-^ - P+ | -0.13 | 0.086 | 203 | -1.513 | 0.132 |

**Table S8.** Model-averaged coefficients and predictor importance for rhizosphere fungal Shannon diversity responses (only P+ samples) at ASV (top panel) and guild (bottom) levels (H2). Estimates are derived from multimodel inference using the ΔAICc ≤ 4 candidate model set. Relative importance **(Σw)** reflects the summed Akaike weights across all models including the predictor. Model count indicates the number of top models in which each predictor was retained. Bold P-values indicate *P* < 0.05.

| ***ASV*** |  |  |  |  |  |  |
| --- | --- | --- | --- | --- | --- | --- |
| **Predictor** | **Estimate (β)** | **Adjusted SE** | **z value** | **P value** | **Relative importance (Σw)** | **Model count** |
| (Intercept) | 1.845 | 0.107 | 17.313 | **<0.001** | — | — |
| Marsh Microbe | 0.824 | 0.110 | 7.505 | **<0.001** | 1 | 4 |
| N Input | -0.147 | 0.109 | 1.351 | 0.177 | 1 | 4 |
| IW | 0.104 | 0.049 | 2.122 | **0.034** | 0.220 | 1 |
| Marsh Microbe × N Input | -0.045 | 0.209 | 0.216 | 0.829 | 0.100 | 1 |
| Age | -0.121 | 0.104 | 1.163 | 0.245 | 0.100 | 1 |
| ***Guild*** |  |  |  |  |  |  |
| **Predictor** | **Estimate (β)** | **Adjusted SE** | **z value** | **P value** | **Relative importance (Σw)** | **Model count** |
| (Intercept) | 0.524 | 0.067 | 7.88 | **<0.001** | — | — |
| Marsh Microbe | 0.502 | 0.064 | 7.866 | **<0.001** | 1 | 2 |
| N Input | 0.028 | 0.062 | 0.447 | 0.655 | 1 | 2 |
| Age Cohort | -0.118 | 0.064 | 1.848 | 0.065 | 0.230 | 1 |

**Table S9.** Structural equation model (SEM) results for fungal diversity (Shannon). Standardized SEM coefficients are shown for ASV-based (top panel) and ecological guild-based (bottom) datasets. Predictors include plant traits and experimental factors: marsh microbe inoculation, N input, and plant age cohort. Values reported are unstandardized estimates, standard errors (SE), test statistics (z), P values, and standardized coefficients (Std. Estimate). Bold P-values indicate *P* < 0.05.

| ***ASV*** |  |  |  |  |  |  |
| --- | --- | --- | --- | --- | --- | --- |
| **Response** | **Predictor** | **Estimate** | **Std.Error** | **Crit.Value** | **P.Value** | **Std.Estimate** |
| RS | IW | 0.052 | 0.119 | 0.435 | 0.665 | 0.040 |
| RS | Marsh Microbe | -0.172 | 0.214 | -0.804 | 0.423 | -0.074 |
| RS | N Input | -0.758 | 0.210 | -3.618 | **<0.001** | -0.335 |
| RS | Age Cohort | -0.526 | 0.219 | -2.408 | **0.018** | -0.223 |
| BG | IW | 0.473 | 0.177 | 2.667 | **0.009** | 0.247 |
| BG | Marsh Microbe | -0.587 | 0.321 | -1.830 | 0.070 | -0.170 |
| BG | N Input | -0.430 | 0.314 | -1.371 | 0.173 | -0.127 |
| BG | Age Cohort | -0.678 | 0.327 | -2.074 | **0.041** | -0.193 |
| SDi | IW | 0.041 | 0.043 | 0.963 | 0.338 | 0.096 |
| SDi | Marsh Microbe | -0.019 | 0.077 | -0.253 | 0.801 | -0.025 |
| SDi | N Input | 0.034 | 0.075 | 0.450 | 0.654 | 0.045 |
| SDi | Age Cohort | 0.034 | 0.078 | 0.438 | 0.663 | 0.044 |
| SLA | IW | -0.086 | 0.091 | -0.942 | 0.349 | -0.092 |
| SLA | Marsh Microbe | -0.097 | 0.165 | -0.590 | 0.556 | -0.058 |
| SLA | N Input | 0.267 | 0.161 | 1.657 | 0.101 | 0.162 |
| SLA | Age Cohort | 0.279 | 0.168 | 1.664 | 0.099 | 0.162 |
| SN | IW | 0.185 | 0.086 | 2.157 | **0.033** | 0.208 |
| SN | Marsh Microbe | -0.303 | 0.155 | -1.954 | 0.054 | -0.188 |
| SN | N Input | 0.082 | 0.152 | 0.539 | 0.591 | 0.052 |
| SN | Age Cohort | 0.062 | 0.158 | 0.393 | 0.695 | 0.038 |
| Shannon | IW | 0.127 | 0.059 | 2.147 | **0.034** | 0.172 |
| Shannon | RS | 0.046 | 0.087 | 0.535 | 0.594 | 0.081 |
| Shannon | BG | -0.045 | 0.087 | -0.519 | 0.605 | -0.117 |
| Shannon | SDi | -0.066 | 0.163 | -0.404 | 0.688 | -0.038 |
| Shannon | SLA | 0.042 | 0.065 | 0.649 | 0.518 | 0.054 |
| Shannon | SN | 0.052 | 0.168 | 0.309 | 0.758 | 0.063 |
| Shannon | Marsh Microbe | 0.827 | 0.105 | 7.868 | **<0.001** | 0.623 |
| Shannon | N Input | -0.150 | 0.108 | -1.398 | 0.165 | -0.116 |
| Shannon | Age Cohort | -0.115 | 0.112 | -1.028 | 0.306 | -0.085 |
| ~~BG | ~~RS | 0.396 | - | 4.351 | **<0.001** | 0.396 |
| ~~SDi | ~~BG | 0.386 | - | 4.231 | **<0.001** | 0.386 |
| ~~SN | ~~BG | 0.756 | - | 11.652 | **<0.001** | 0.756 |
| ~~SLA | ~~SDi | 0.293 | - | 3.097 | **0.001** | 0.293 |
| ~~SN | ~~SLA | 0.186 | - | 1.915 | **0.029** | 0.186 |
| ~~SN | ~~SDi | 0.527 | - | 6.260 | **<0.001** | 0.527 |
| ~~SDi | ~~RS | -0.169 | - | -1.736 | **0.043** | -0.169 |
| ***Guild*** |  |  |  |  |  |  |
| **Response** | **Predictor** | **Estimate** | **Std.Error** | **Crit.Value** | **P.Value** | **Std.Estimate** |
| RS | IW | 0.052 | 0.119 | 0.435 | 0.665 | 0.040 |
| RS | Marsh Microbe | -0.172 | 0.214 | -0.804 | 0.423 | -0.074 |
| RS | N Input | -0.758 | 0.210 | -3.618 | **<0.001** | -0.335 |
| RS | Age Cohort | -0.526 | 0.219 | -2.408 | **0.018** | -0.223 |
| BG | IW | 0.473 | 0.177 | 2.667 | **0.009** | 0.247 |
| BG | Marsh Microbe | -0.587 | 0.321 | -1.830 | 0.070 | -0.170 |
| BG | N Input | -0.430 | 0.314 | -1.371 | 0.173 | -0.127 |
| BG | Age Cohort | -0.678 | 0.327 | -2.074 | **0.041** | -0.193 |
| SDi | IW | 0.041 | 0.043 | 0.963 | 0.338 | 0.096 |
| SDi | Marsh Microbe | -0.019 | 0.077 | -0.253 | 0.801 | -0.025 |
| SDi | N Input | 0.034 | 0.075 | 0.450 | 0.654 | 0.045 |
| SDi | Age Cohort | 0.034 | 0.078 | 0.438 | 0.663 | 0.044 |
| SLA | IW | -0.086 | 0.091 | -0.942 | 0.349 | -0.092 |
| SLA | Marsh Microbe | -0.097 | 0.165 | -0.590 | 0.556 | -0.058 |
| SLA | N Input | 0.267 | 0.161 | 1.657 | 0.101 | 0.162 |
| SLA | Age Cohort | 0.279 | 0.168 | 1.664 | 0.099 | 0.162 |
| SN | IW | 0.185 | 0.086 | 2.157 | **0.033** | 0.208 |
| SN | Marsh Microbe | -0.303 | 0.155 | -1.954 | 0.054 | -0.188 |
| SN | N Input | 0.082 | 0.152 | 0.539 | 0.591 | 0.052 |
| SN | Age Cohort | 0.062 | 0.158 | 0.393 | 0.695 | 0.038 |
| Shannon | IW | 0.050 | 0.036 | 1.382 | 0.170 | 0.112 |
| Shannon | RS | 0.054 | 0.053 | 1.015 | 0.313 | 0.155 |
| Shannon | BG | -0.063 | 0.053 | -1.198 | 0.234 | -0.272 |
| Shannon | SDi | -0.151 | 0.100 | -1.520 | 0.132 | -0.144 |
| Shannon | SLA | 0.025 | 0.040 | 0.634 | 0.528 | 0.053 |
| Shannon | SN | 0.116 | 0.103 | 1.121 | 0.265 | 0.231 |
| Shannon | Marsh Microbe | 0.513 | 0.064 | 7.967 | **<0.001** | 0.636 |
| Shannon | N Input | 0.026 | 0.066 | 0.397 | 0.692 | 0.033 |
| Shannon | Age Cohort | -0.134 | 0.069 | -1.955 | 0.054 | -0.164 |
| ~~BG | ~~RS | 0.396 | - | 4.351 | **<0.001** | 0.396 |
| ~~SDi | ~~BG | 0.386 | - | 4.231 | **<0.001** | 0.386 |
| ~~SN | ~~BG | 0.756 | - | 11.652 | **<0.001** | 0.756 |
| ~~SLA | ~~SDi | 0.293 | - | 3.097 | **0.001** | 0.293 |
| ~~SN | ~~SLA | 0.186 | - | 1.915 | **0.029** | 0.186 |
| ~~SN | ~~SDi | 0.527 | - | 6.260 | **<0.001** | 0.527 |
| ~~SDi | ~~RS | -0.169 | - | -1.736 | **0.043** | -0.169 |
